# DNA damage repair gene mutations and their association with tumor immune regulatory gene expression in muscle invasive bladder cancer subtypes

**DOI:** 10.1186/s40425-019-0619-8

**Published:** 2019-06-07

**Authors:** Thiago Vidotto, Sarah Nersesian, Charles Graham, D. Robert Siemens, Madhuri Koti

**Affiliations:** 1 Genetics Department, Medicine School of Ribeirão Preto, University of São Paulo, Ribeirão Preto, Brazil; 2 Department of Biomedical and Molecular Sciences and Obstetrics and Gynecology, Queen’s University, K7L3N6, Kingston, Ontario Canada; 3 Department of Urology, Queen’s University, Kingston, Ontario Canada; 4 Cancer Biology and Genetics, Queen’s Cancer Research Institute, Kingston, Ontario Canada; 5 Department of Obstetrics and Gynecology, Kingston, Ontario Canada

**Keywords:** Bladder Cancer, Interferon, DNA damage repair, Immune checkpoint

## Abstract

**Background:**

Molecular subtyping of urothelial cancer (UC) has significantly advanced the understanding of bladder tumor heterogeneity and development of prognostic and predictive biomarkers. Evolving evidence across cancers strongly suggests that tumor immunoediting has a profound impact on the behaviour of cancer cells and their adaptation to the co-evolving microenvironment and response to treatment. In alignment with these concepts, recent immune checkpoint blockade (ICB) therapies in UC have demonstrated the predictive potential of mutations in the DNA damage repair (DDR) genes. A comprehensive understanding of DDR gene inactivation associated expression of immune regulatory genes could thus aid in expansion of current immunotherapies and predictive biomarkers for the design of patient-tailored combination treatments.

**Methods:**

We investigated pre-treatment tumor transcriptomic profiles of the five recently described molecular subtypes of muscle invasive urothelial cancer (MIUC; *n* = 408) from The Cancer Genome Atlas, to determine subtype specific immune cell abundance, expression of 67 immune regulatory genes, and association with DDR gene inactivation (via mutation, copy number alteration) profiles.

**Results:**

Analysis using CIBERSORT immune cell abundance determination tool showed significant differences in immune cell profiles and abundance between MIUC subtypes. Expression patterns of a selected panel of 67 genes including both immune stimulatory and inhibitory genes, showed significant associations with subtypes, and DDR gene mutation status.

**Conclusion:**

Findings from our study provide compelling evidence for co-expression of multiple immune checkpoint genes including, *PD-1*, *PD-L1, IDO1, TIGIT, TIM-3, TGFB1, LAG3,* and others, that potentially contribute to compensatory immune evasion in bladder tumors. Our findings also emphasize the urgent need for biomarker discovery approaches that combine molecular subtype, DDR gene mutation status, tumor immune landscape classification, and immune checkpoint gene expression to increase the number of patients responding to immunotherapies.

**Electronic supplementary material:**

The online version of this article (10.1186/s40425-019-0619-8) contains supplementary material, which is available to authorized users.

## Introduction

The optimal management of non-metastatic muscle invasive urothelial cancer (MIUC) incorporates cystectomy, extended lymph node dissection, and peri-operative chemotherapy for those able to tolerate such intensive regimens. For those presenting or progressing to metastatic disease, palliative cisplatin-based chemotherapy is utilized but with limited response rates and generally poor overall improvement in survival up to 5–6% at 5 years [1]. The presence of high tumor mutational burden in urothelial cancers (UC) confers high immunogenicity, which makes these tumors good candidates for immunotherapy strategies, such as immune checkpoint blockade (ICB) [2]. Indeed, ICB therapy targeting the programmed death ligand-1 (PD-L1)/PD-1 immune checkpoint axis has shown some promising results in the treatment of UC; however, only a small subset of patients with metastatic disease had durable responses [3–5]. The major hurdles in achieving optimal survival benefits from ICB include tumor intrinsic heterogeneity, variations in pre-treatment tumor immune contexture, lack of predictive biomarkers, and optimal drug sequencing strategies. There is an urgent need to develop treatment combinations to enhance the proportion of chemotherapy and ICB responsive patients.

Several molecular classification schemes have been reported for MIUC subtyping, opening opportunities towards patient stratification for subtype tailored treatments [6]. There is a consensus that UC can be broadly divided into basal and luminal subtypes, which show distinct prognosis and response to chemotherapy [7]. Through in silico immune transcriptomic profiling, we recently reported that the four MIUC clusters earlier defined by The Cancer Genome Atlas (TCGA) network exhibit distinct immune gene expression patterns [8]. Robertson et al., 2017 reported the presence of five molecular subtypes in the TCGA MIUC cohort, a newer classification scheme, which incorporated the neuronal mRNA subtype [6].

The classification for treatment naïve immune contexture, which is critical in the context of current immunotherapy, divides solid tumors into “**T cell-inflamed or hot**” or “**T cell non-inflamed or cold**” categories [9]. An immunologically hot tumor is characterized by higher expression of IFN genes and corresponding higher density of **activated** CD8^+^ tumor infiltrating lymphocytes (TILs) [10]. Cold tumors usually show higher levels of immunosuppressive genes, lower density of activated CD8^+^ TILs and increased FoxP3^+^ or regulatory populations of immune cells. In the context of MIUC, luminal tumors are generally immunologically cold (with the exception of ‘luminal infiltrated’ subtype). It is intriguing that patients with luminal tumors show an increased overall survival, which is contradictory to their cold tumor state [11]. In contrast, patients with basal MIUC tumors exhibiting a hot tumor state have poorer outcomes, albeit their increased sensitivity to chemotherapy. The precise mechanisms underlying these counterintuitive associations remain to be fully elucidated although adaptive immune resistance mechanisms could be one of the potential underlying factors.

One interesting mechanism that regulates cellular Type I IFN responses, is the loss of function mutations in DNA damage repair (DDR) genes [12–18]. Approximately 40% of MIUC exhibit mutations in DDR genes [6]. It is widely established that pre-treatment breast (basal) and ovarian tumors with DDR gene (*BRCA1/2*) mutations are immunologically hot with high CD8^+^ TILs [19–21]. Moreover, these tumors exhibit increased response to platinum-based chemotherapy and have longer progression-free survival [22, 23]. Higher mutational burden leading to more neo-antigens in tumors with DDR deficiency has been suggested as one of the mechanisms that triggers spontaneous TIL infiltration in breast and ovarian cancers [21, 24]. This cancer agnostic association between DDR deficiency and treatment response is also seen in MIUC [21, 25–27]. Several reports in UCs have confirmed the strong association between mutations in DDR genes including, *ERCC2, ATM*, *FANCD2*, *PALB2*, *BRCA1*, *BRCA2, RB1* and sensitivity to platinum-based neoadjuvant chemotherapy and chemoradiation therapy [28–33]. Most recently, Teo et al. confirmed that approximately 47% of advanced/metastatic UCs have at least one DDR gene mutation and exhibited higher sensitivity to platinum based chemotherapy [26].

A comprehensive view of the DDR mutation associated pre-treatment immune landscape is currently not available for MIUC. This knowledge is key to the future design of biomarker guided immunotherapy treatment combinations. To test our hypothesis that pre-treatment immune contexture and subsequent response of MIUC is potentially dictated by cancer cell intrinsic events such as DDR deficiency, we interrogated the subtype specific expression profiles of a panel of **“immune regulatory”** (immune-stimulatory and immune-inhibitory/checkpoint) genes using whole transcriptomic data from the TCGA (*n* = 408). We further correlated the expression profiles of immune regulatory genes with most prevalent DDR gene mutations in MIUC.

## Methods

### In silico immune-cell density analysis of MIUC RNA-Seq data

We downloaded raw and level 3 RNAseq, array-CGH (aCGH), SNV, and associated clinical data of 408 MIUC tumors from the updated TCGA data (https://portal.gdc.cancer.gov/). Whole PanCancer-normalized transcriptome profiles were employed to classify each sample using the previously described five bladder cancer mRNA subtypes [6]. For quantification of immune cell abundance in each molecular subtype, we performed an in silico deconvolution of 22 immune cell types through the CIBERSORT algorithm (https://cibersort.stanford.edu) [34].

We also investigated the associations between the MIUC molecular subtypes for the abundance of T helper type (Th) 1, Th2, and Th17 immune cells. Data for these cell types were obtained from a published study from the TCGA group [35]. The immune-cell scores were then compared between the molecular subtypes through Kruskal-Wallis test. *P*-values below 0.05 indicated significant differences between groups.

Based on CIBERSORT relative scores for all 22 immune cell types, we dichotomized the abundance of the CD8+ T cells levels as high (≥ average) and low (< average) to determine their impact on the overall and recurrence-free survival of patients. The dichotomized score was applied in log-rank tests and generation of Kaplan-Meier curves for each MIUC subtype.

### Characterization of DDR gene inactivation status

To identify if there is an association between MIUC subtypes, DDR gene alterations and immune response gene expression patterns, we conducted an integrative analysis using gene expression, mutation and copy number alteration status, and immune cell abundance using all bladder tumor profiles from the TCGA cohort. We selected the most prevalent DDR genes that exhibit loss via mutations or copy number alterations in genes associated with DDR in MIUC based on recent literature as well as their known association with cellular IFN responses [6]. To determine their inactivation status, we combined copy number calls with the presence of somatic inactivating mutations (non-synonymous mutations). Genes presenting a hemizygous loss or one single somatic point mutation in one allele were classified as undergoing monoallelic inactivation. The concomitant presence of a hemizygous deletion and a somatic point mutation in the remaining allele characterized genes with biallelic inactivation. The presence of homozygous deletions was also classified as biallelic inactivation. The presence of gene amplifications and concomitant point mutations in one allele or no copy number alterations were classified as having no effect on protein function and expression.

### Analysis of subtype specific expression of 67 immune-regulatory genes

To investigate whether there is a subtype specific differential expression of immune-regulatory genes across the MIUC subtypes, we investigated the expression of a panel of 67 immune-regulatory (consisting of both immune activators and immune inhibitors; Additional file 1: Table S1) genes that are either associated with immune stimulation or inhibition in the tumor microenvironment [36]. The correlation between the immune-regulatory gene expression scores and the 22 immune cell types was determined by Spearman’s correlation test. Similarly, the correlation between the expression of DDR genes and immune cells and immune-regulatory genes was assessed with Spearman’s correlation test. For survival analysis, we used the recurrence-free survival and overall survival data to perform log-rank analyses through the *survival* package in R Bioconductor. *P*-values below 0.05 were considered as statistically significant.

**Table 1:** Subtype specific abundance of CD8+ TILs in MIUC. Abundance of CD8+ TILs in 408 MIUC tumors was determined using CIBERSORT tool. High and low CD8+ TIL groups were defined based on the average relative score obtained from CIBERSORT output. Relative scores above and below the average were classified as high and low CD8+ TIL abundance, respectively

Subtype	CD8+ TIL high	CD8+ TIL low	Total
Basal squamous	55	87	142
Luminal	7	19	26
Luminal infiltrated	25	53	78
Luminal papillary	40	102	142
Neuronal	4	16	20
Number of cases	131	277	408

### Associations between DDR gene inactivation status and immunogenic mutations

To determine if DDR gene inactivation and MIUC molecular subtype are associated with genomic changes that may drive an anti-tumor immune response, we downloaded Level 3 tumor purity and immunogenic somatic mutation data from a published study from the TCGA group [35]. Immunogenic mutations were determined based on the number of point mutations that coded for neoantigens. We investigated the tumor mutational burden by analyzing the rates of non-synonymous mutations and also determined the impact of DDR inactivation and MIUC subtype in the presence of immunogenic mutations.

### Correlation between DDR gene mutation and immune-regulatory gene expression

To visualize gene expression patterns, we generated heatmaps using the ComplexHeatmap package in R v3.4.3. We then obtained the average expression z-scores per MIUC subtype for the 67 genes investigated. In addition, we obtained the average CIBERSORT score per MIUC subtype for the 22 immune cells. Similar calculations were performed for samples with mutations in each of the DDR genes. The gene expression and immune cell profiles were compared for each of the DDR gene mutations and MIUC subtypes through Kruskal-Wallis test.

## Results

### MIUC tumors exhibit mRNA subtype specific immune cell infiltration patterns

**Fig. 1:**
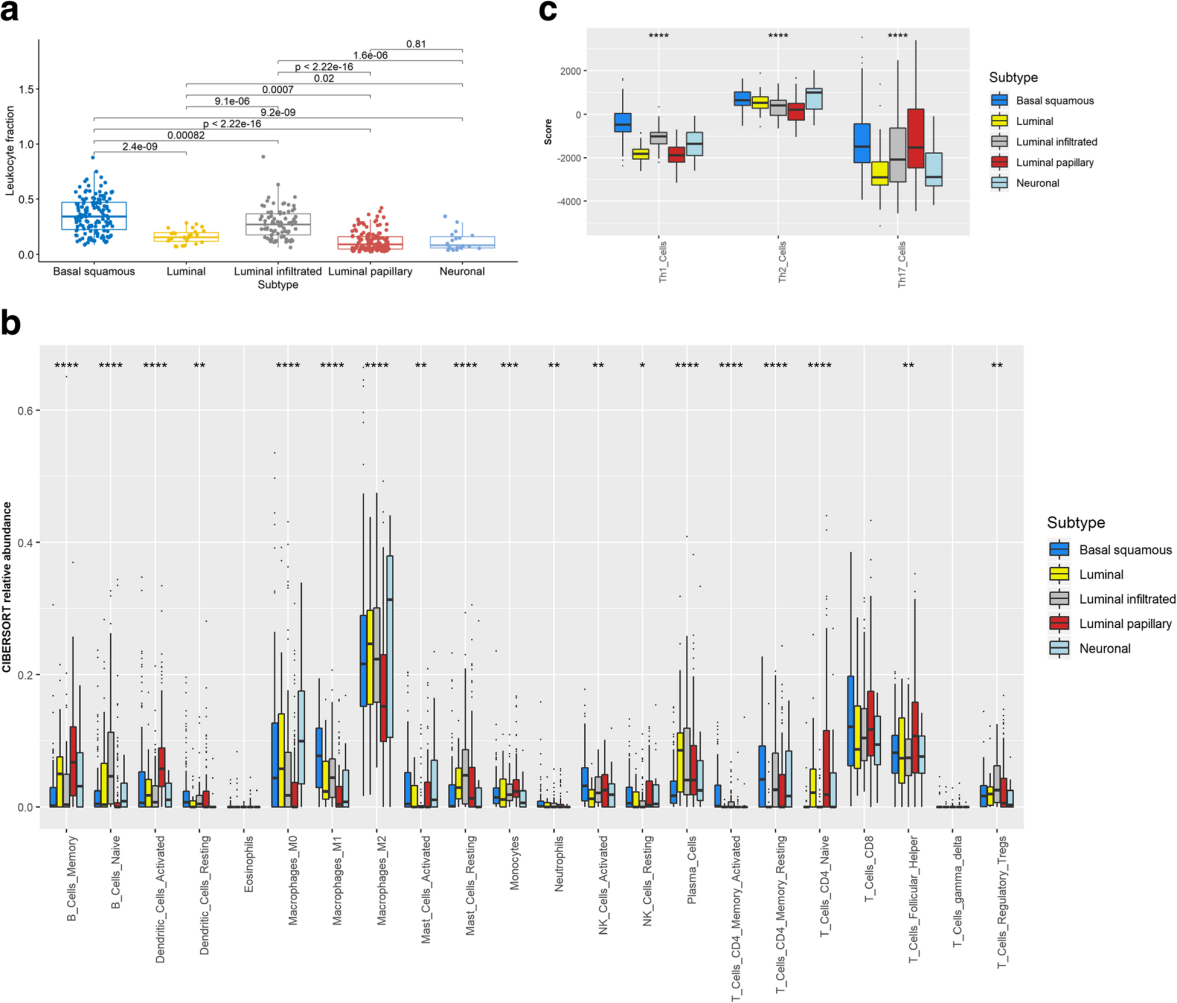
Subtype specific immune cell abundance in pre-treatment MIUC tumors. ABSOLUTE tumor purity score and CIBERSORT-derived immune cell relative scores were used to determine the abundance of immune cells in the five bladder cancer subtypes. Basal squamous tumors followed by luminal infiltrated showed the highest levels of leukocyte fractions (**a**). Significantly higher abundance of CD8+ T-cells, activated memory CD4+ T-cells, and M1 macrophages was observed in tumors classified as basal squamous subtype (**b**). Luminal papillary tumors exhibit high abundance of naïve CD4+ T-cell, memory B-cells, follicular helper T-cells, and active dendritic cells (**b**). We also found that basal squamous tumors exhibit Th1 responses within the TME (**c**). Comparisons were performed by employing Kruskal-Wallis test. *P < 0.05, **P < 0.01, ***P < 0.001, ****P < 0.0001

In concordance with previous findings, we observed that basal squamous and luminal infiltrated subtypes are T-cell inflamed/hot tumors, while neuronal, luminal, and luminal papillary tumors are immunologically cold (Kruskal-Wallis test, *P* < 0.05) (Fig. 1b). CIBERSORT-based analysis revealed that basal squamous tumors have significantly higher natural killer (NK) cells, M1 macrophage, and memory CD4^+^ T cell abundance compared to other subtypes (Kruskal-Wallis test, *P* < 0.01) (Fig. 1b). Neuronal tumors also showed the lowest abundance of regulatory T cells (Treg) in contrast with luminal infiltrated and luminal tumors. A significantly higher abundance of memory B cells, T follicular helper cells, and active dendritic cells was also seen in the luminal papillary tumors (Kruskal-Wallis test, *P* < 0.01). These findings suggest that basal subtype tumors are heavily immune infiltrated with features of a T helper type I (Th1) immune response. Indeed, this observation was confirmed after our analysis of the abundance of Th1, Th2, and Th17 immune cells per subtype (Fig. 1c).

Since there are distinct profiles of immune cells within the TME of the MIUC molecular subtypes, we hypothesized that the immune profile of these groups was potentially a consequence of immunogenic features, such as expressed neoantigens. We thus compared the number of immunogenic mutations and did not identify significant differences between subtypes (Additional file 1: Figure S1, Kruskall-Wallis test, *P* > 0.05). Concordantly, the subtypes shared similar number of non-synonymous mutations (Additional file 2: Figure S1, Kruskall-Wallis test, *P* > 0.05). We then investigated whether chromosomal instability as seen by genome doubling events (referred to as ploidy) were linked to the differences seen in immune-cell profiles across subtypes. Luminal papillary and basal squamous had the lowest levels of ploidy, while only luminal papillary showed high levels of tumor purity (Additional file 2: Figure S2, Kruskall-Wallis test, *P* < 0.05).

### Immune-regulatory gene expression profiles associate with MIUC mRNA subtypes

**Fig. 2:**
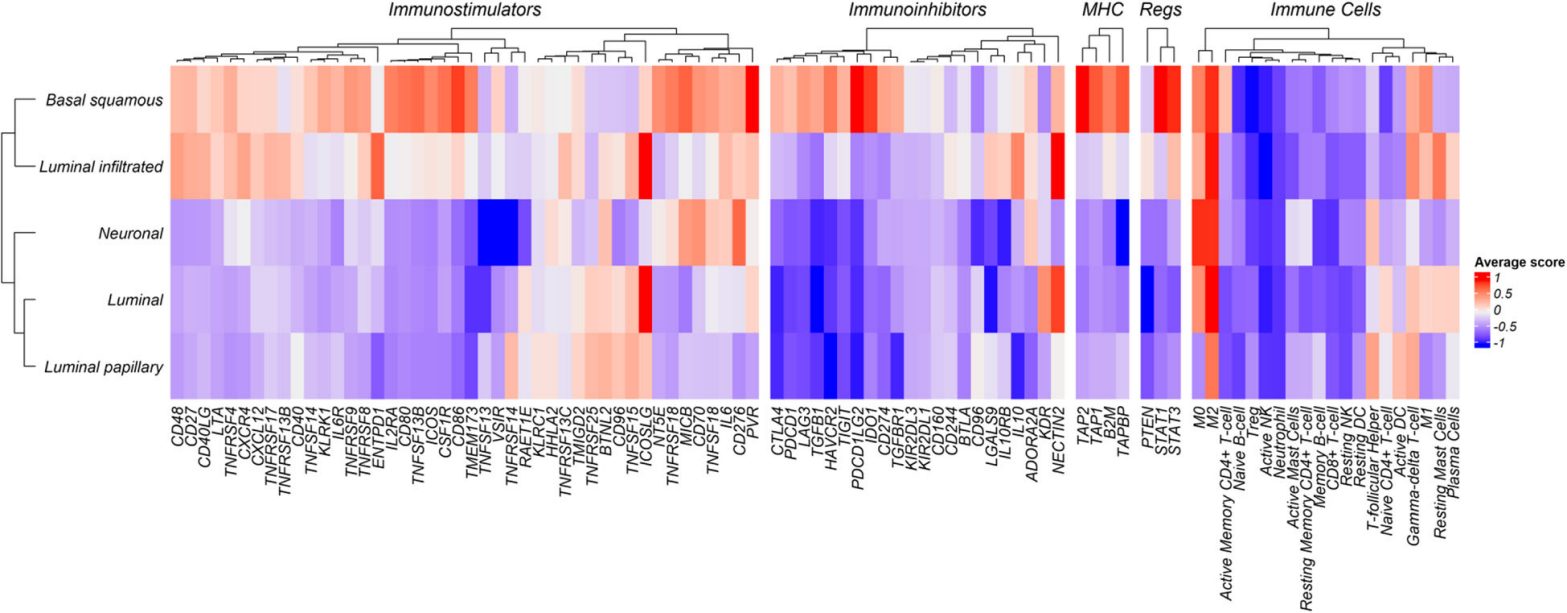
MIUC subtypes exhibit distinct expression patterns of immunoregulatory genes. Average z-score of each immune-regulatory gene was obtained per subtype (row). Unsupervised clustering show clustering of basal squamous and luminal infiltrated tumors with higher scores for immunostimulatory genes. Basal squamous showed high expression scores of immunoinhibitors, such as CTLA4, IDO1, TIGIT, LAG3, PD-1, and PD-L1 compared with other subtypes

Since we did not identify significant associations between overall genomic changes and the immune-cell composition between MIUC subtypes, we investigated whether alterations in immune gene expression had an impact or were related to the presence of immune cells in the TME. Analysis of a panel of 67 immune-regulatory genes, including both immune activators and inhibitors, showed a distinct association with the MIUC molecular subtypes (Fig. 2). Basal squamous and luminal infiltrated subtypes were part of a shared cluster with high expression of immune inhibitors, MHC-associated genes, and *STAT1* and *STAT3* transcription factors (Fig. 2, Additional file 2: Figure S3). Interestingly, the expression of *PD-L1, CTLA-4, IDO1, LAG3, ICOS, MICB, STAT1,* and *STAT3* in basal squamous tumors was significantly higher than the expression in other molecular subtypes (Additional file 2: Figures S3a and S3b, Kruskal-Wallis test, *P* < 0.05).

### MIUC with high pre-existing CD8^+^ TILs exhibit features of adaptive immune resistance

**Fig. 3:**
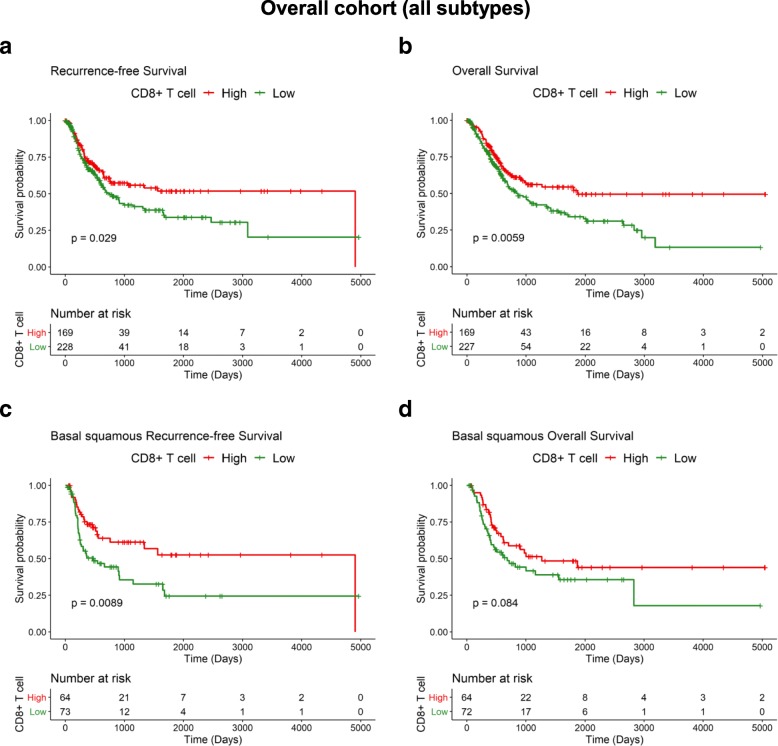
High CD8+ TIL abundance associates with decreased recurrence in MIUC The relative immune cell scoring for CD8+ T-cells were dichotomized as high and low to determine its impact on bladder cancer outcome. Low CD8+ TIL abundance significantly associated with earlier disease recurrence (**a**) in a subset of 318 patients with available recurrence data. The association between CD8+ T-cell abundance and overall survival (**b**) was also significant. Basal squamous tumors showed two distinct groups of high and low CD8+ T-cell abundance with a significant association with disease recurrence within (**c**) and a trend for significance in predicting shorter overall survival for this MIUC subtype (**d**)

Given the prognostic relevance of CD8^+^ TILs in solid tumors, we dichotomized the 408 pre-treatment MIUC into CD8^+^ TIL high and low groups. Interestingly, CD8^+^ TIL abundance was highly variable among the five subtypes (Table 1). Survival analysis of all 408 tumors demonstrated that low CD8^+^ TILs were linked with shorter recurrence-free and overall survival (log-rank test, *P* = 0.029 and *P* = 0.0059, respectively) (Fig. 3a and b). For basal squamous tumors, the population with low CD8+ TIL had poor outcome, as depicted by shorter recurrence-free survival and a similar trend in overall survival (log-rank test, *P* = 0.008 and *P* = 0.08, respectively) (Fig. 2c and d). We did not observe significant associations between CD8+ TIL abundance and outcome for the other four MIUC molecular subtypes (log-rank, *P* > 0.05) (Additional file 2: Figure S4).

**Fig. 4:**
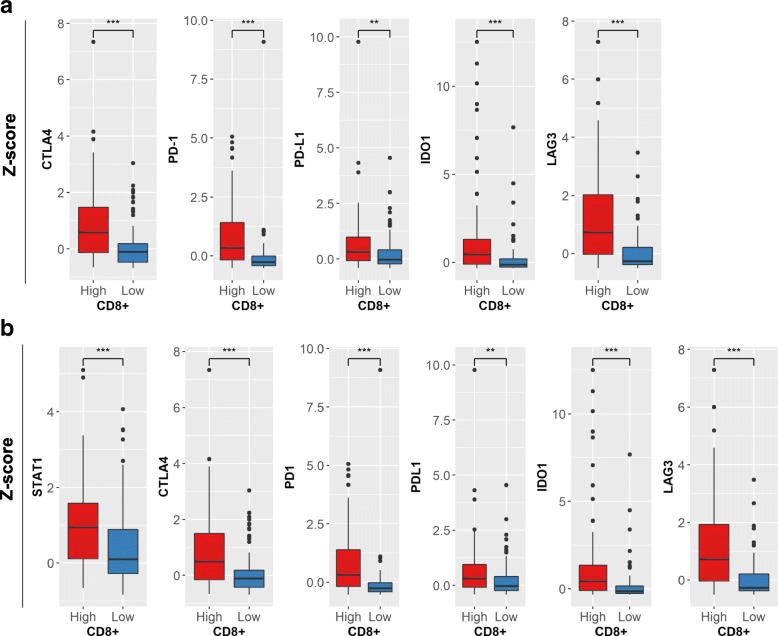
MIUC tumors with high CD8+ TILs show significantly increased expression of immune checkpoint genes in overall cohort (**a**) and basal squamous subtype (**b**). Y-axis demonstrates z-scored expression values. Comparisons were performed by employing Mann-Whitney test. *P < 0.05, **P < 0.01, ***P < 0.001

We then compared the expression profiles of immune regulatory genes in the 408 tumors with high vs. low CD8^+^ TILs. Tumors with high CD8^+^ TIL showed significantly higher levels of immune checkpoint genes *CTLA4, PD-1, PD-L1, IDO1,* and *LAG3* compared to the low CD8+ TIL group (Mann-Whitney test, *P* < 0.01) (Fig. 4a). Further, when we stratified by molecular subtype, basal squamous tumors with high CD8+ T-cells also showed high levels of the above mentioned immune checkpoint genes, in addition to *STAT1* (Fig. 4b). This novel finding of increased immune checkpoint gene expression in the basal squamous subtype indicates the existence of IFN induced adaptive immune resistance, a potential factor contributing to an aggressive disease.

### Mutations in DDR genes associate with immunoregulatory gene expression profiles

Since overall genomic and chromosomal instability failed to explain the differences in the immune-cell composition within each MIUC subtype, we aimed to investigate the effects of DDR mutations in the anti-tumor activity in the TME. Recent reports have provided evidence for a role of DDR mutations in cellular IFN responses. Subtype specific evaluation of DDR mutation frequencies are shown in Additional file 2: Figure S5. Notably, *ATM*, *RB1*, and *TP53* were among the most inactive DDR genes (via biallelic loss, Additional file 2: Figure S5a, S5b, S5c). In an unsupervised clustering, monoallelic inactivation of *ATM*, *RB1*, and *TP53* were part of the same group and showed high expression of *STAT3*, but only tumors with *TP53*-monoallelic  inactivation had high average levels of *CD70* and *IL6* (Additional file 2: Figure S7). Intact DDR genes did not show distinct patterns of immunoregulatory gene expression (Additional file 2: Figure S6).

**Fig. 5:**
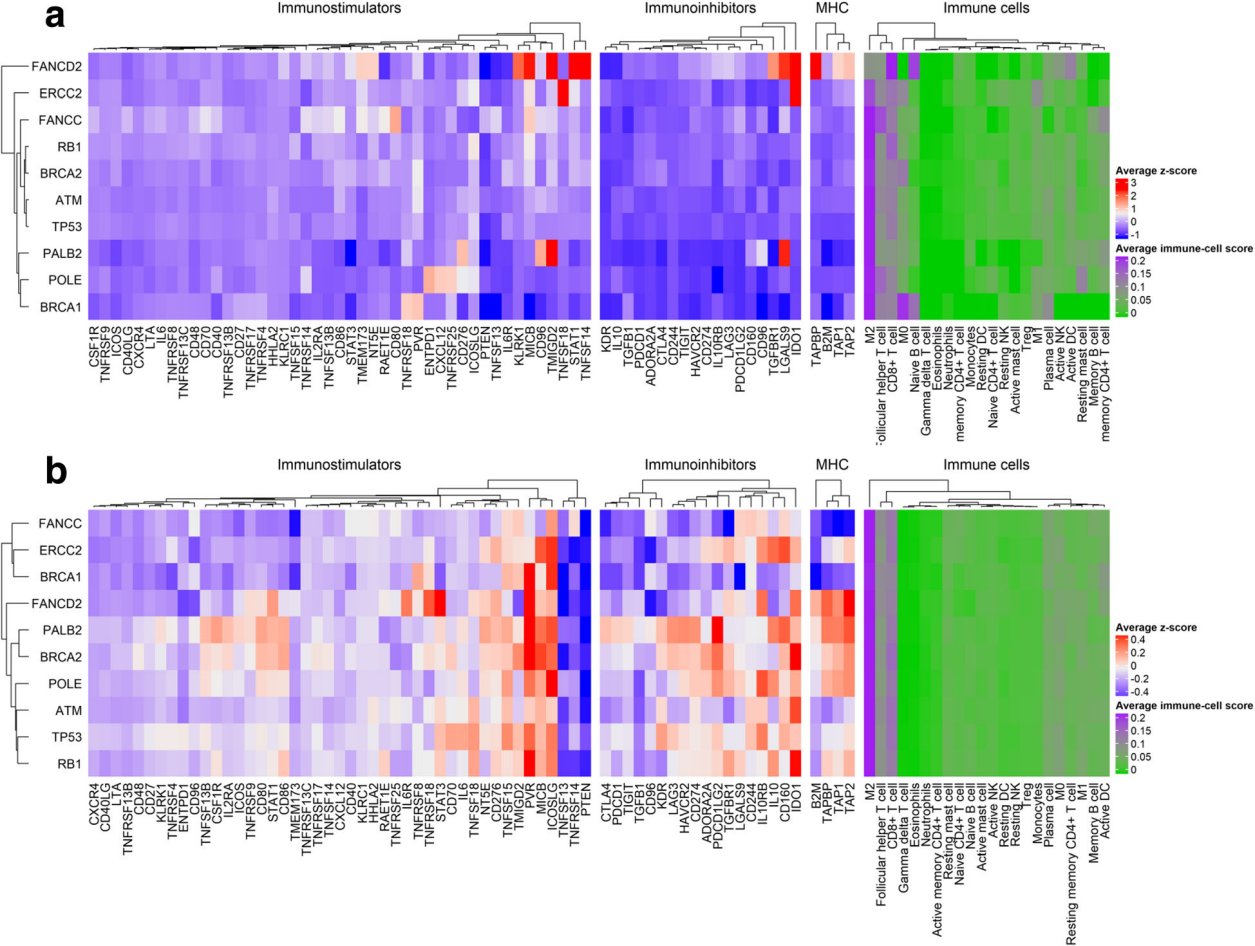
DDR mutations associate with immune regulatory gene expression in MIUC. Tumors with DDR mutations show differences in expression patterns of immunoregulatory genes. The effect of DDR biallelic and monoallelic inactivation in the expression of immune-regulatory genes are shown in (**a**) and (**b**), respectively. There is a gradual decrease in the average expression levels of tumors harboring DDR mutations. In contrast, wild-type tumors exhibit high levels of expression across tumors (d Additional file 1: Fig. S6). The average z-score was obtained for each investigated DDR gene (averaged z-score by row). Mutations in DDR genes in the TCGA MIUC were not mutually exclusive with the presence of concurrent mutations

Comparison between wild-type vs. mutated *TP53* tumors showed that *TP53* biallelic mutations significantly associate with high expression of *KDR* (Kruskal-Wallis test, *P* < 0.001) (Additional file 2: Figure S7). Interestingly, *TP53* biallelic mutations were also strongly associated with low *PTEN* and *STING* expression (Additional file 2: Figure S6, Kruskal-Wallis test, *P* < 0.01). Also, we found that *RB1* biallelic inactivation was linked to higher *PD-L1* expression (Additional file 2: Figure S7, Kruskal-Wallis text, *P* < 0.001). By averaging z-score expression levels per tumors harboring either biallelic, monoallelic, or wild-type mutations in the DDR genes, we observed that biallelic tumors exhibit lower expression of immune-regulatory genes compared with other DDR status (Fig. 5).

We found the expression of DDR genes negatively correlated with the expression of immune-regulatory genes (Spearman correlation test, *P* < 0.05) (Additional file 2: Figure S8). In contrast, *ATM* expression was positively correlated with *CD28, IL2RA, CD80*, and *IL6*. When stratified by subtype, we observed a distinct pattern of gene expression correlation and CD8+ TIL abundance for each group. Neuronal, luminal, and luminal infiltrated tumors exhibited strong negative correlations between genes (Spearman correlation test, *P* < 0.05, Additional file 2: Figure S9). In contrast, luminal papillary and basal squamous tumors – which presented with the highest levels of immune-cell infiltration – demonstrated strong positive correlations (Spearman correlation test, *P* < 0.05, Additional file 2: Figure S9).

To determine the potential role of DDR gene mutation on the immunogenicity of tumors, we analysed the impact of DDR inactivation in the levels of immunogenic mutations and Treg and CD8+ TIL abundance. We found that biallelic mutations in *ATM*, *BRCA1/2*, *PALB2*, *RB1*, and *TP53* were linked to significant increase in immunogenic mutations (Additional file 2: Figure S10, Kruskal-Wallis test, *P* < 0.01). However, no significant association was found when comparing the presence of cytolytic and regulatory cells within the TME of MIUC tumors (Additional file 2: Figure S10, Kruskal-Wallis test, *P* > 0.05).

## Discussion

Recent success of immune checkpoint blockade therapies has re-directed the focus of molecular subtyping in MIUC to obtaining a deeper understanding of the cellular and secreted factors of the TME and its evolution by cancer cell intrinsic genomic alterations. Bladder cancer is amongst the few solid tumors where durable responses from ICB treatment have been observed in a subset of patients. A wide array of ICB trials targeting the PD-1/PD-L1 and CTLA-4 immune checkpoints are in progress with combinations using both adjuvant and neo-adjuvant chemotherapy in metastatic UC [37]. There is an urgent need to increase the proportion of patients responding to ICB. This can only be accomplished via the development of robust predictive biomarkers and superior combinatorial treatment approaches.

Amongst the cancers affecting the genitourinary system such as prostate and renal cell carcinoma, the bladder tumor immune contexture appears distinct, with higher mutational burden and features ranging from absence of TILs (immune desert) to a highly infiltrated tumor (immunologically hot) [37–39]. Relevant to our study findings, are the results of the recently completed ICB trials including IMvigor210 and Check Mate 275 trials that demonstrated the efficacy of PD-L1 and PD-1 inhibitors in platinum refractory MIUC patients [4]. Upon subtyping using the TCGA MIUC subtypes, these trials confirmed that tumors belonging to the luminal subtype and those lacking PD-L1 expression did not respond to atezolizumab (PD-L1 inhibitor). Another key finding from this trial was the cancer cell-specific expression of PD-L1 exclusively seen in tumors belonging to the TCGA basal subtype, which did not correlate with objective response rate [4]. These findings are suggestive of diverse impacts of immune cell versus cancer cell-specific expression of PD-L1 on ICB response.

Our analyses revealed the highest expression of immune checkpoint genes including *CTLA-4, LAG3, TIGIT, PD-1, PD-L1, IDO1, TGFB, TGFBR1, TIM-3* and others in the basal squamous tumors. Based on these findings it can be speculated that an ICB treatment unresponsive tumor state is either due to lack of target expression or due to increased PD-L1 expression that imparts aggressive properties to cancer cells via activation of cellular proliferation pathways such as ERK and mTOR [40]. Our speculation is supported by previous findings, including our report, that showed the cancer cell intrinsic role of PD-L1 in mediating drug resistance, autophagy, and activation of aggressive pathways [40–42]. Within the basal MIUC tumors, it is also possible that immunogenic cell death induced by chemotherapy leads to enhanced tumor antigen cross-presentation to the pre-existing TILs leading to increased chemosensitivity. Activated TILs producing IFN-γ could further induce the expression of immune checkpoint genes with subsequent evolution of aggressive disease phenotype. These observations are also suggestive of counter-regulatory mechanisms in cancer cells (via possibly simultaneous expression of immune checkpoints PD-L1 and IDO1) putatively driven by genetic defects such as DDR mutations, that could eventually modulate the response to ICB [10]. Based on similar rationale, several ongoing trials in solid tumors are investigating the combinatorial effect of PARP inhibitors with immune checkpoint blockade [43]. Given the co-expression of immune checkpoint genes, predictive biomarker studies should also include evaluating simultaneous expression of proteins such as TIGIT, IDO1, LAG3, TIM-3 and others in addition to PD-L1.

Surprisingly, the results of KEYNOTE-045 Phase III trial using PD-1 targeting pembrolizumab, did not show a correlation between responses and tumor PD-L1 expression status [44]. Indeed, the high TGFβ expression in tumors leading to immune exclusion may not be discounted, which is also evident from our findings. This notion is supported by the recent report by Mariathasan et al., which showed tumors from aetozolizumab non-responsive patients had higher expression of *TGFβ* and its receptors and lacked CD8^+^ TILs in the tumor epithelial compartment [45]. Characterization of MIUC via subtyping prior to treatment and introduction of TGFβ inhibitors or immunostimulatory agents such as IFN inducing drugs, could thus potentially sensitize these tumors to ICB.

The outcome of CheckMate275 Phase II trial evaluating nivolumab (PD-1 inhibitor) in 270 UC patients, confirmed higher expression of a 25 gene IFN-γ signature in tumors from responders [3]. The most important and critical finding from this study, is the significantly higher expression of IFN activated *STAT1*, its downstream target TIL recruiting chemokine genes, *CXCL9, CXCL10, CXCL11* in addition to the checkpoint molecules *IDO1, LAG3, PD-1*, markers of CD8^+^ T cell activation such as *PRF1, GZMB* and *CD8A* in a subset of basal tumors [3]. Interestingly, the treatment responsive group of patients was also enriched in a subgroup of basal subtype. Our findings in the basal squamous subtype and the presence of a group with CD8^+^ high TILs and high expression of *PD-1, LAG-3, IDO1, CTLA-4* and *PD-L1* showing better prognosis and decreased disease recurrence, are in concordance with this finding. Interestingly, basal breast cancer molecular subtypes that overlap in classification schemes applied in bladder, also exhibit features of higher chemosensitivity, presence of two basal subgroups and higher frequency of mutations in DDR genes and higher immune infiltrates [46, 47]. Notably, these features are also common in serous ovarian cancer where DDR deficiency associates with high immune activity in the TME [48]. A cancer type agnostic concurrent evolution of adaptive immune resistance due to increased immune checkpoint gene expression in these tumors, could potentially be a factor contributing to overall poor prognosis in basal UCs. Future chemo-immunotherapy trials should thus use combined subtyping by immunohistochemistry and immune checkpoint gene expression assay based biomarker signatures as tools for patient selection.

DDR mutation status of tumors is an important predictive biomarker of ICB response [45]. It is now well established that cancers with DDR mutations are immunologically hot and responsive to ICB. Three recent trials in UC including, NCT02553642, NCT01928394, and NCT02108652 (www.clinicaltrials.gov) have confirmed that mutations in DDR genes, are predictors of response to PD-1/PD-L1 ICB [27, 45]. Furthermore, it is now widely established that cancer cells with DDR deficiency exhibit constitutive activation of cellular IFN responses and secretion of TIL recruiting chemokines, CCL5 and CXCL10 [25, 49]. DDR mutation status of a tumor could also be combined with complementary biomarker assays for patient selection for ICB. Based on evolving concepts on the activation of cytosolic nucleic acid sensing IFN activating pathways such as cGAS-STING or RIG that ultimately lead to production of chemokines CCL5 and CXCL10 [50], it is possible that spontaneous TIL infiltration occurs simultaneous to malignant progression of tumors with DDR deficiency leading to an immunologically hot pre-treatment state. Although mechanistic studies are warranted to establish these associations further, our findings revealed increased expression patterns of the key IFN mediators *STAT1* and/or *STAT3* in tumors with DDR mutations such as those in *ATM, ERCC1, RB1, BRCA2, POLE* and *TP53*, reflective of IFN pathway activation.

**Fig. 6:**
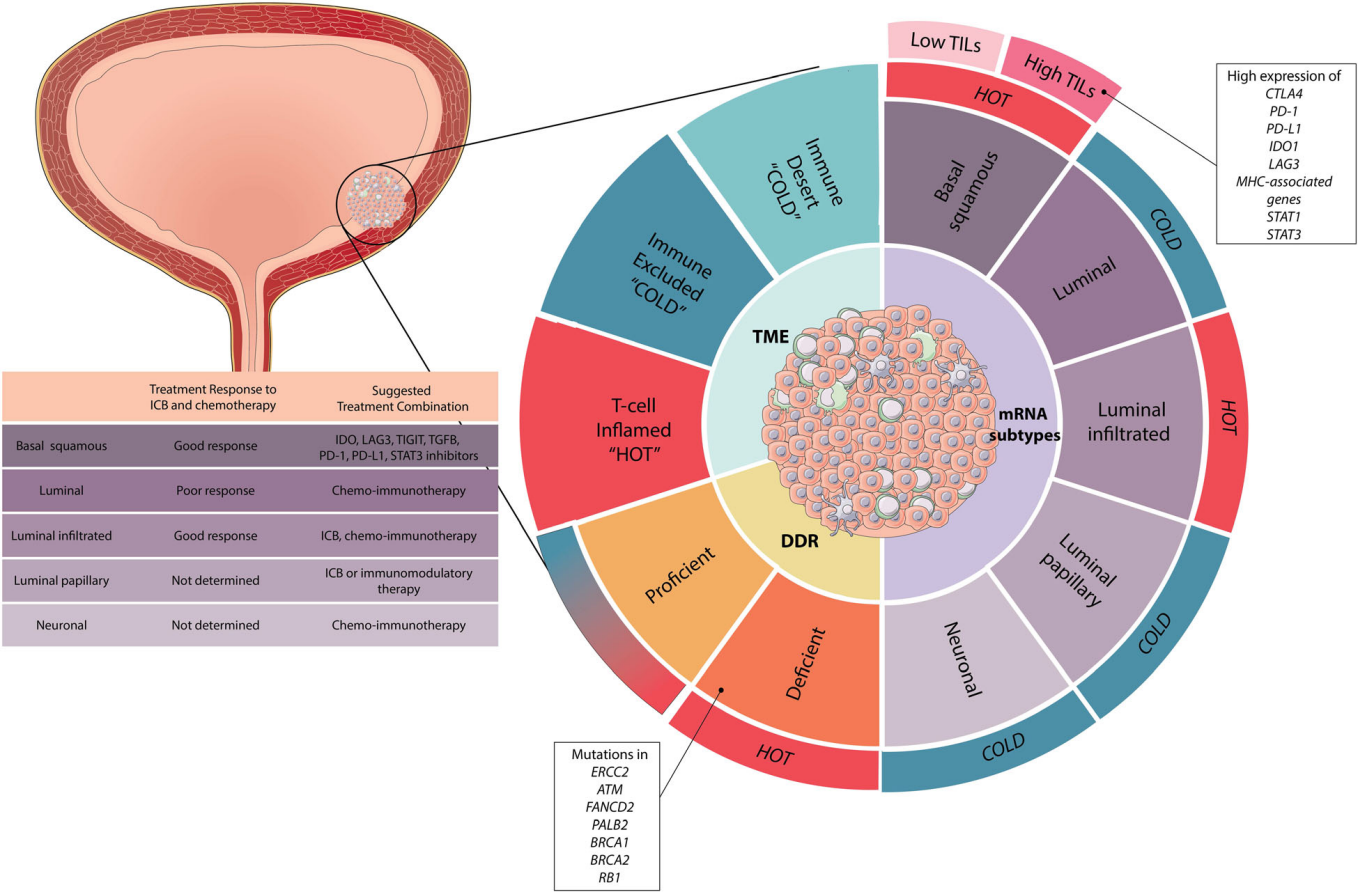
Proposed scheme for combination biomarkers to stratify MIUC patients for chemo-immunotherapy. Tumor mRNA subtype, DDR mutation status and immunoregulatory gene expression levels should be included in one combination biomarker assay to select patients for chemo-immunotherapy. Image design by http://www.designsthatcell.ca/

Regardless of the mechanisms of evolution, in the context of current ICB treatments, information on DDR mutation status combined with assays to determine cancer and immune cell specific expression of immune checkpoint expression could further allow rational design of combinatorial immunotherapy trials. In summary (Fig. 6), the recent large and partially successful ICB trial outcomes re-emphasize the urgent need for the development of novel biomarker guided and combinatorial immunomodulatory treatments that can be used for MIUC patients with or without standard systemic chemotherapies. Given the development to novel immune stimulatory agents and the significance of DDR in activating cytosolic innate sensing pathways, these agents could be incorporated in treatment regimes, to sensitize MIUC tumors to ICB [51].

A limitation of our study is the lack of information on spatial organization of immune cell profiles in the five molecular subtypes of MIUC. Integrative analysis of genomic and transcriptomic alterations combined with the evaluation of spatial organization of TILs and immune checkpoint proteins within the tumor core compared to invasive margins is absolutely critical for development of an immune classifier for patient selection for conventional chemotherapy as well as for ICB or other immunomodulatory therapies. Single-cell sequencing approaches should also be performed on cancer cells isolated from pre-treatment tumors to investigate the presence of multiregional diversity or spatial heterogeneity or presence of multiple clones with variability in immune-regulatory gene expression patterns and associated immune profiles. Another factor that was demonstrated as predictive biomarker for ICB response, is the tumor mutational burden [45]. In our study we did not evaluate the correlation between TMB and its correlation with subtypes, DDR status and immune contexture. Previous reports in other cancers have indeed, confirmed an association between DDR mutations and high TMB, increased chemosensitivity and higher TILs.

## Conclusion

In conclusion, our results are suggest the potential co-activation of multiple compensatory immune checkpoint pathways in pre-treatment MIUC and thus provide rationale for use of combination ICB treatment. Findings from our comprehensive analyses will aid in the rational design of subtype specific combination immunomodulatory treatment approaches in UC.

## Additional files

Additional file 1: **Supplementary Table 1**. List of the 67 immune-regulatory genes. (DOCX 12 kb)

Additional file 2: **Figure S1.** Number of non-synonymous mutations by MIUC subtypes. **Figure S2.** Immune and stromal scores demonstrate no difference in the immune content between bladder cancer subtypes. **Figure S3.** Subtype associated immunoregulatory gene expression profile in MIUC. **Figure S4.** Association between CD8+ TIL and survival in MIUC subtypes. **Figure S5a.** Frequency of DDR gene inactivation by somatic mutation and copy number alterations. Dupl –duplication, Nef –No effect on protein expression. **Figure S5b.** Subtype associated frequency of DDR gene inactivation by somatic mutation and copy number alterations. **Figure S5c.** Effect of biallelic inactivation of DDR genes in the expression of immunomodulators. **Figure S6.** Comparison between wild-type vs. mutated TP53 tumors showed that TP53 biallelic mutations significantly associate with high expression. **Figure S7.** Correlation between expression of immunoregulatory and DDR genes in MIUC, identified by spearman correlation analysis. **Figure S8.** Correlation between expression of immunoregulatory and DDR genes in MIUC subtypes, identified by spearman correlation analysis. **Figure S9.** DDR gene mutations associate with immunogenic mutations and abundance of CD8+ TILs and Tregs. **Figure S10.** Association between DDR inactivation and levels of immunogenic mutations, Treg and CD8+ TIL abundance, ploidy and purity. (PPTX 3186 kb)
