# Supplementary material for: DNA damage repair gene mutations and their association with tumor immune regulatory gene expression in muscle invasive bladder cancer subtypes
Source: J Immunother Cancer. 2019 Jun 7;7:148. doi: 10.1186/s40425-019-0619-8 (PMC6556053; doi:10.1186/s40425-019-0619-8)
Supplement: Supplementary file 1 — Supplementary Table 1. List of the 67 immune-regulatory genes. (DOCX 12 kb) [file 40425_2019_619_MOESM1_ESM.docx]

| Stimulators | *CD27, CD276, CD40, CD40LG, CD48, CD70, CD80, CD86, CD96, CSF1R, CXCL12, CXCR4, ENTPD1, HHLA2, ICOS, ICOSLG, IL2RA, IL6, IL6R, KLRC1, KLRK1, LTA, MICB, NT5E, PVR, PTEN, STAT1, STAT3, RAET1E, TMEM173, TMIGD2, TNFRSF13B, TNFRSF13C, TNFRSF14, TNFRSF17, TNFRSF18, TNFRSF25, TNFRSF4, TNFRSF8, TNFRSF9, TNFSF13, TNFSF13B, TNFSF14, TNFSF15, TNFSF18* |
| --- | --- |
| Inhibitors | *ADORA2A, CD160, CD244, CD274, CD96, CTLA4, HAVCR2, IDO1, IL10, IL10RB, KDR, LAG3, LGALS9, PDCD1, PDCD1LG2, TGFB1, TGFBR1, TIGIT* |
| MHC pathway related genes | *B2M, TAP1, TAP2, TAPBP* |

Supplementary Table 1. List of the 67 immune-regulatory genes.
