# Supplementary material for: DNA damage repair gene mutations and their association with tumor immune regulatory gene expression in muscle invasive bladder cancer subtypes
Source: J Immunother Cancer. 2019 Jun 7;7:148. doi: 10.1186/s40425-019-0619-8 (PMC6556053; doi:10.1186/s40425-019-0619-8)
Supplement: Supplementary file 2 — Figure S1. Number of non-synonymous mutations by MIUC subtypes. Figure S2. Immune and stromal scores demonstrate no difference in the immune content between bladder cancer subtypes. Figure S3. Subtype associated immunoregulatory gene expression profile in MIUC. Figure S4. Association between CD8+ TIL and survival in MIUC subtypes. Figure S5a. Frequency of DDR gene inactivation by somatic mutation and copy number alterations. Dupl –duplication, Nef –No effect on protein expression. Figure S5b. Subtype associated frequency of DDR gene inactivation by somatic mutation and copy number alterations. Figure S5c. Effect of biallelic inactivation of DDR genes in the expression of immunomodulators. Figure S6. Comparison between wild-type vs. mutated TP53 tumors showed that TP53 biallelic mutations significantly associate with high expression. Figure S7. Correlation between expression of immunoregulatory and DDR genes in MIUC, identified by spearman correlation analysis. Figure S8. Correlation between expression of immunoregulatory and DDR genes in MIUC subtypes, identified by spearman correlation analysis. Figure S9. DDR gene mutations associate with immunogenic mutations and abundance of CD8+ TILs and Tregs. Figure S10. Association between DDR inactivation and levels of immunogenic mutations, Treg and CD8+ TIL abundance, ploidy and purity. (PPTX 3186 kb) [file 40425_2019_619_MOESM2_ESM.pptx]

## Slide 1
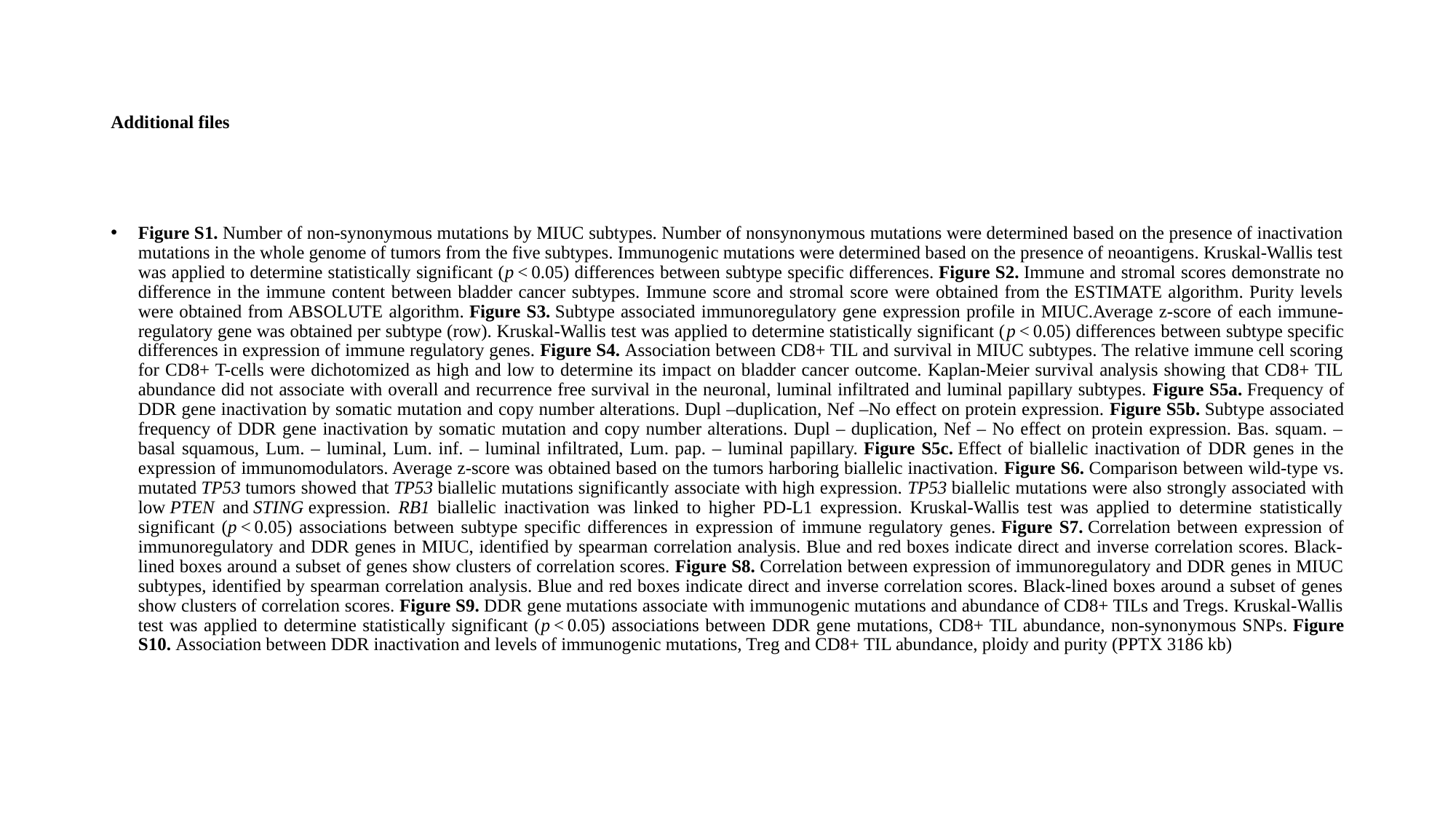

# Additional files
Figure S1. Number of non-synonymous mutations by MIUC subtypes. Number of nonsynonymous mutations were determined based on the presence of inactivation mutations in the whole genome of tumors from the five subtypes. Immunogenic mutations were determined based on the presence of neoantigens. Kruskal-Wallis test was applied to determine statistically significant (p < 0.05) differences between subtype specific differences. Figure S2. Immune and stromal scores demonstrate no difference in the immune content between bladder cancer subtypes. Immune score and stromal score were obtained from the ESTIMATE algorithm. Purity levels were obtained from ABSOLUTE algorithm. Figure S3. Subtype associated immunoregulatory gene expression profile in MIUC.Average z-score of each immune-regulatory gene was obtained per subtype (row). Kruskal-Wallis test was applied to determine statistically significant (p < 0.05) differences between subtype specific differences in expression of immune regulatory genes. Figure S4. Association between CD8+ TIL and survival in MIUC subtypes. The relative immune cell scoring for CD8+ T-cells were dichotomized as high and low to determine its impact on bladder cancer outcome. Kaplan-Meier survival analysis showing that CD8+ TIL abundance did not associate with overall and recurrence free survival in the neuronal, luminal infiltrated and luminal papillary subtypes. Figure S5a. Frequency of DDR gene inactivation by somatic mutation and copy number alterations. Dupl –duplication, Nef –No effect on protein expression. Figure S5b. Subtype associated frequency of DDR gene inactivation by somatic mutation and copy number alterations. Dupl – duplication, Nef – No effect on protein expression. Bas. squam. – basal squamous, Lum. – luminal, Lum. inf. – luminal infiltrated, Lum. pap. – luminal papillary. Figure S5c. Effect of biallelic inactivation of DDR genes in the expression of immunomodulators. Average z-score was obtained based on the tumors harboring biallelic inactivation. Figure S6. Comparison between wild-type vs. mutated TP53 tumors showed that TP53 biallelic mutations significantly associate with high expression. TP53 biallelic mutations were also strongly associated with low PTEN and STING expression. RB1 biallelic inactivation was linked to higher PD-L1 expression. Kruskal-Wallis test was applied to determine statistically significant (p < 0.05) associations between subtype specific differences in expression of immune regulatory genes. Figure S7. Correlation between expression of immunoregulatory and DDR genes in MIUC, identified by spearman correlation analysis. Blue and red boxes indicate direct and inverse correlation scores. Black-lined boxes around a subset of genes show clusters of correlation scores. Figure S8. Correlation between expression of immunoregulatory and DDR genes in MIUC subtypes, identified by spearman correlation analysis. Blue and red boxes indicate direct and inverse correlation scores. Black-lined boxes around a subset of genes show clusters of correlation scores. Figure S9. DDR gene mutations associate with immunogenic mutations and abundance of CD8+ TILs and Tregs. Kruskal-Wallis test was applied to determine statistically significant (p < 0.05) associations between DDR gene mutations, CD8+ TIL abundance, non-synonymous SNPs. Figure S10. Association between DDR inactivation and levels of immunogenic mutations, Treg and CD8+ TIL abundance, ploidy and purity (PPTX 3186 kb)

## Slide 2
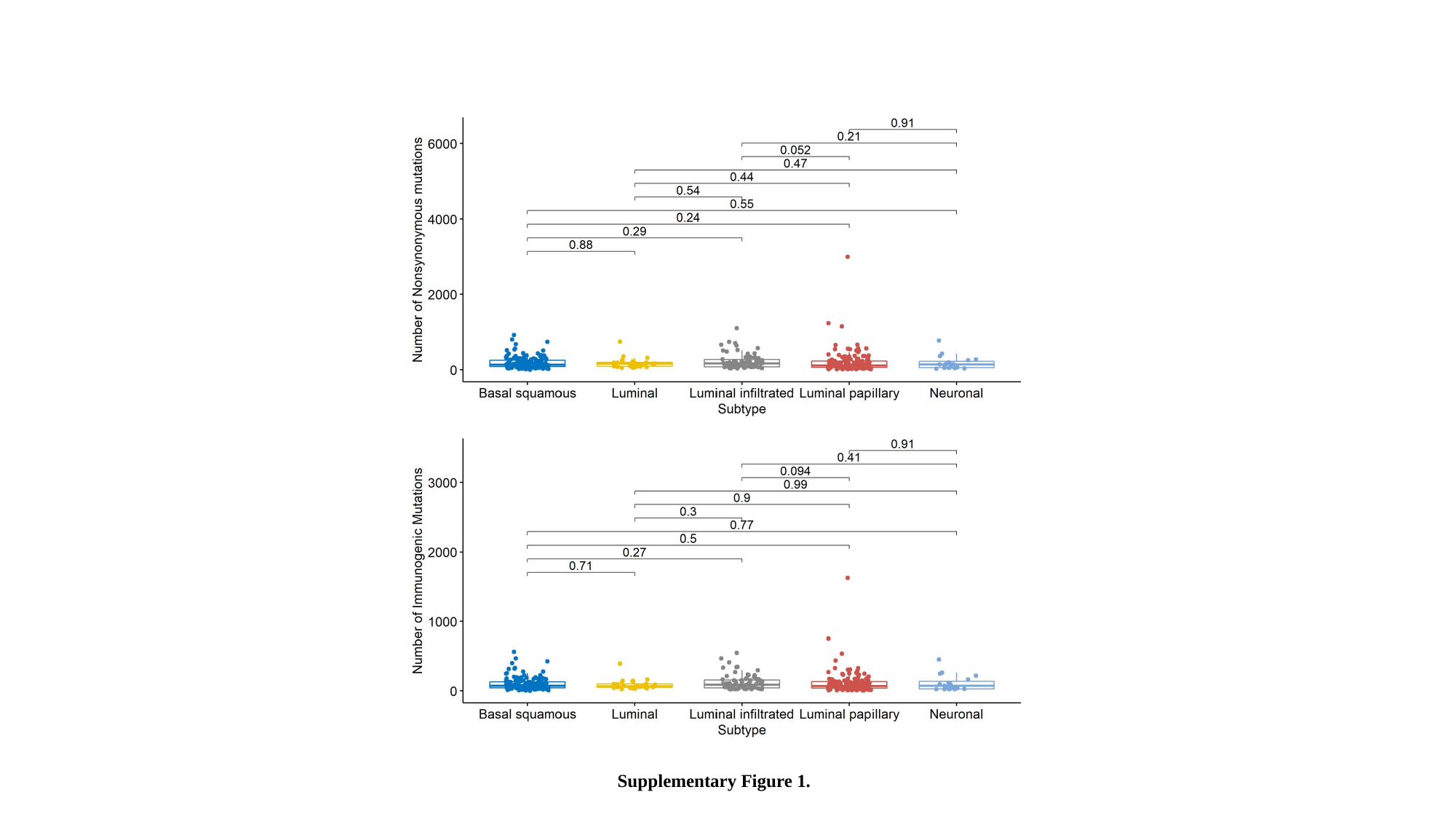

Supplementary Figure 1.

## Slide 3
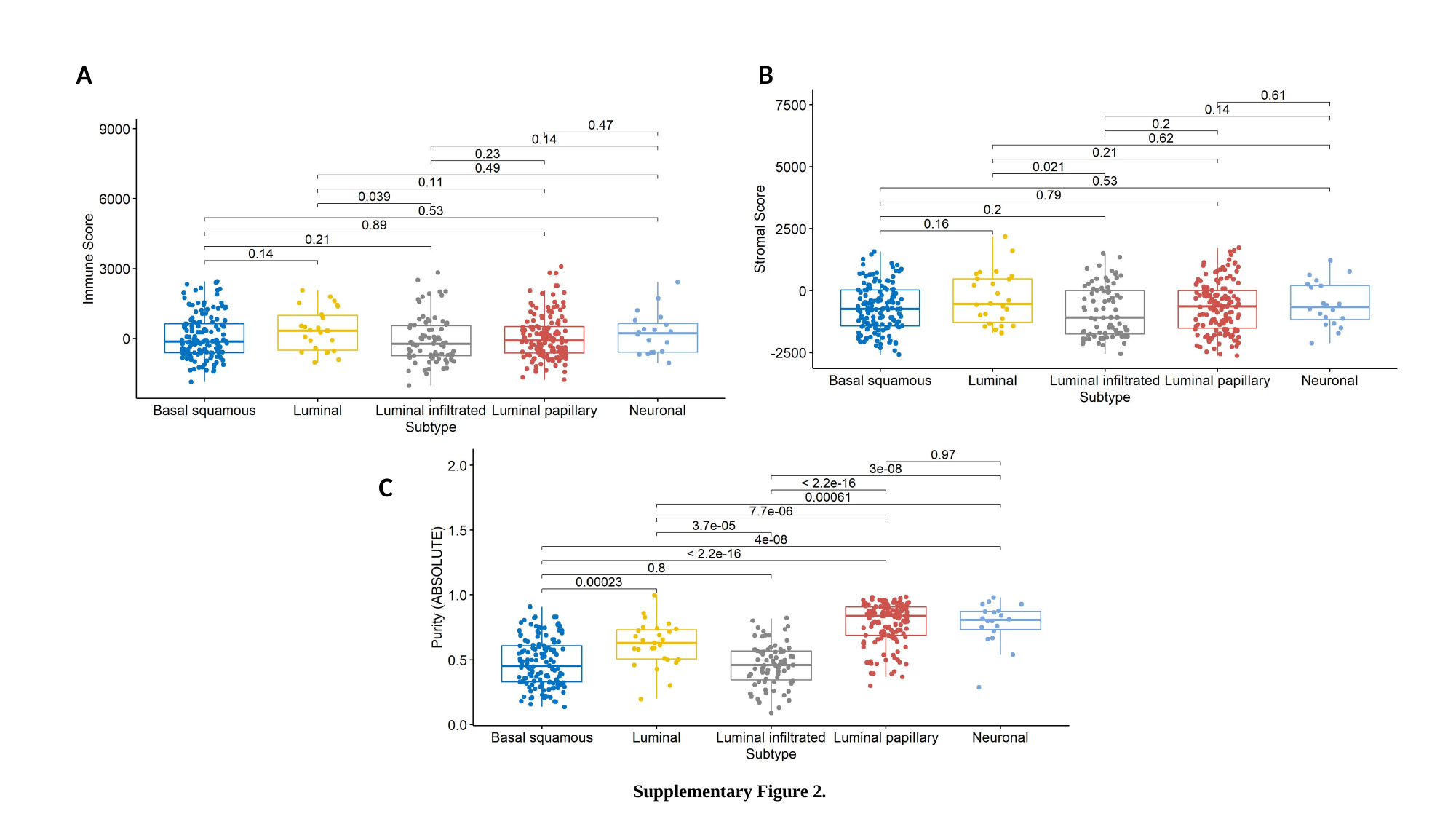

A
B
C
Supplementary Figure 2.

## Slide 4
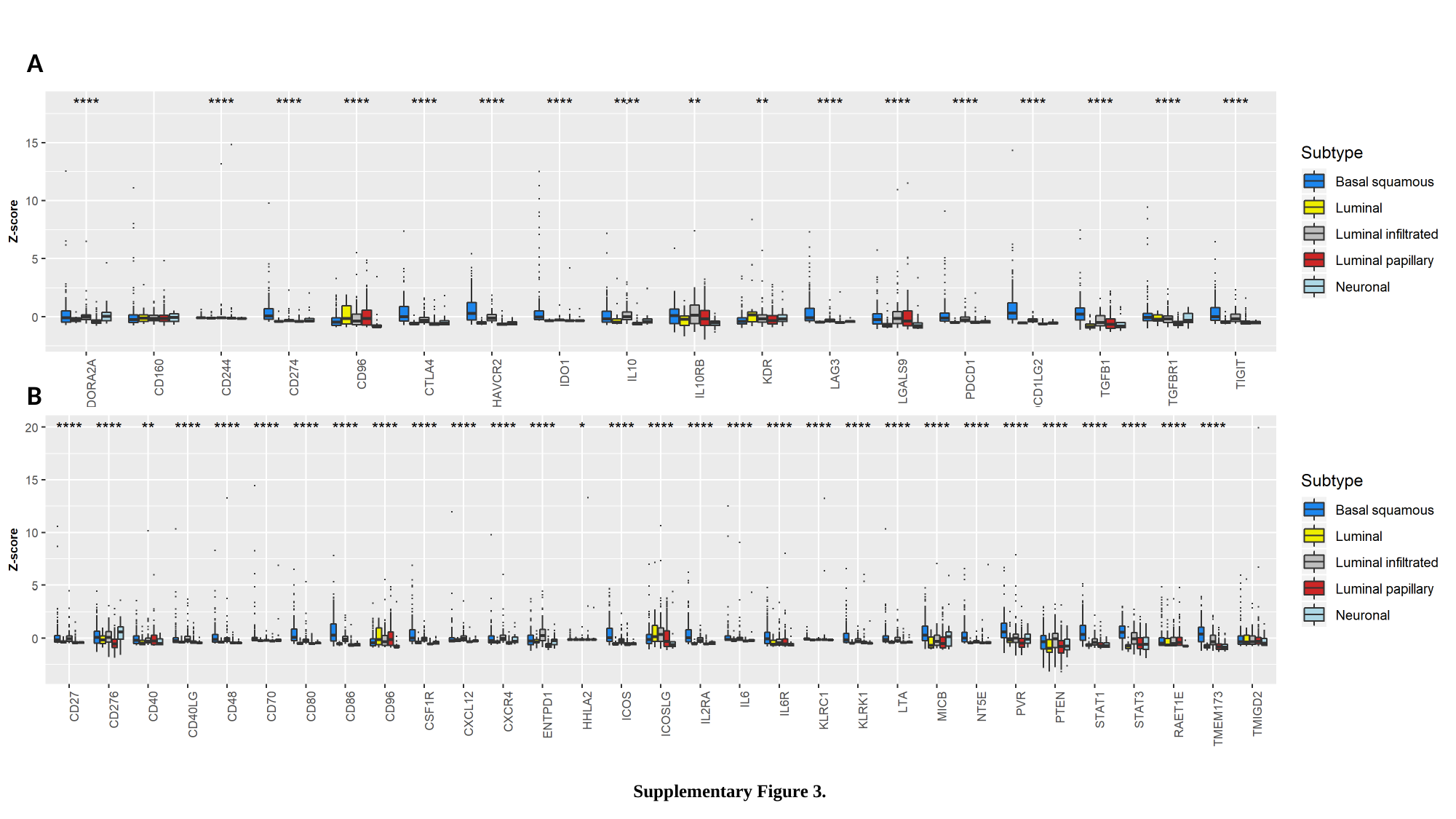

A
B
Supplementary Figure 3.

## Slide 5
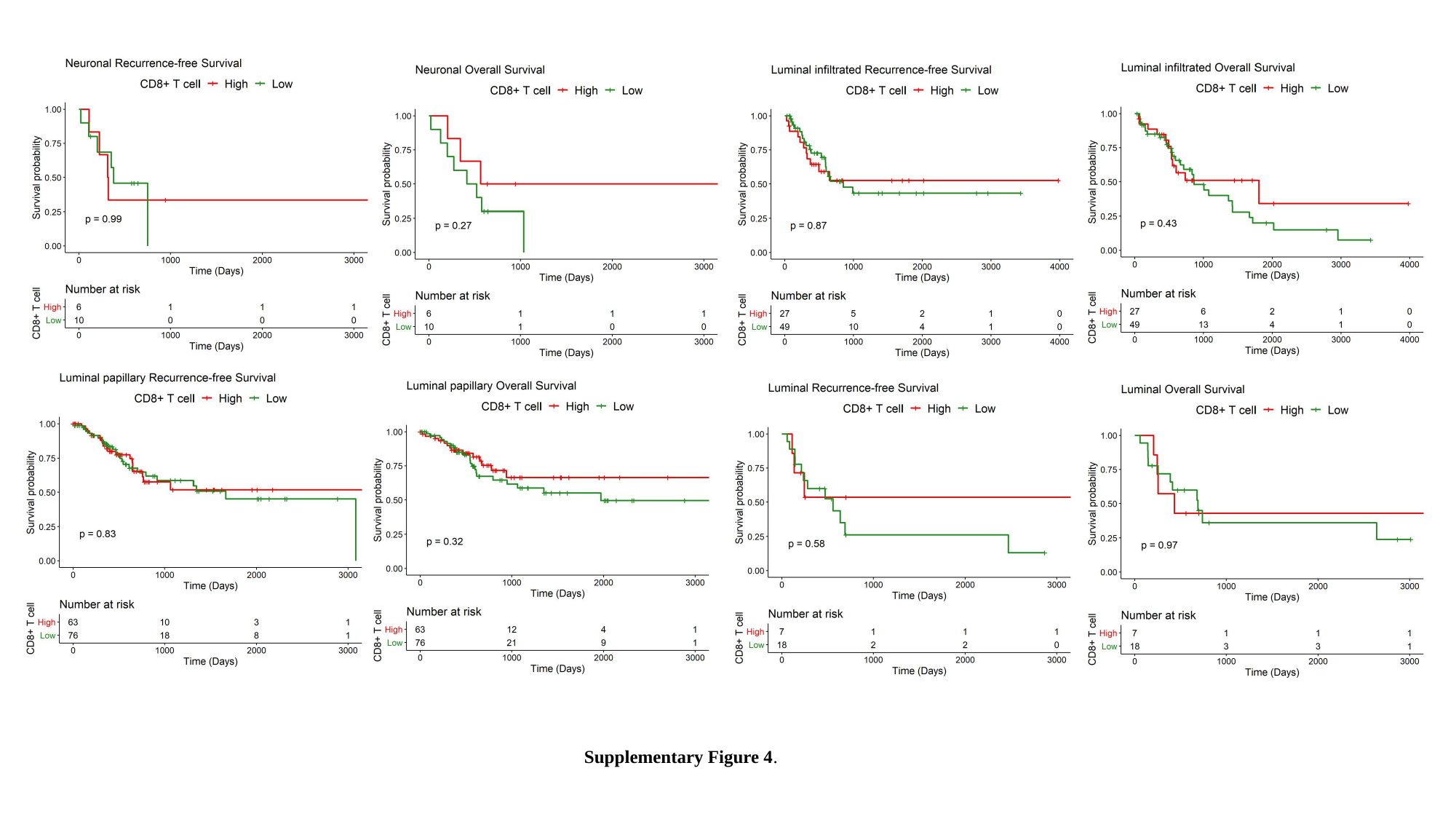

Supplementary Figure 4.

## Slide 6
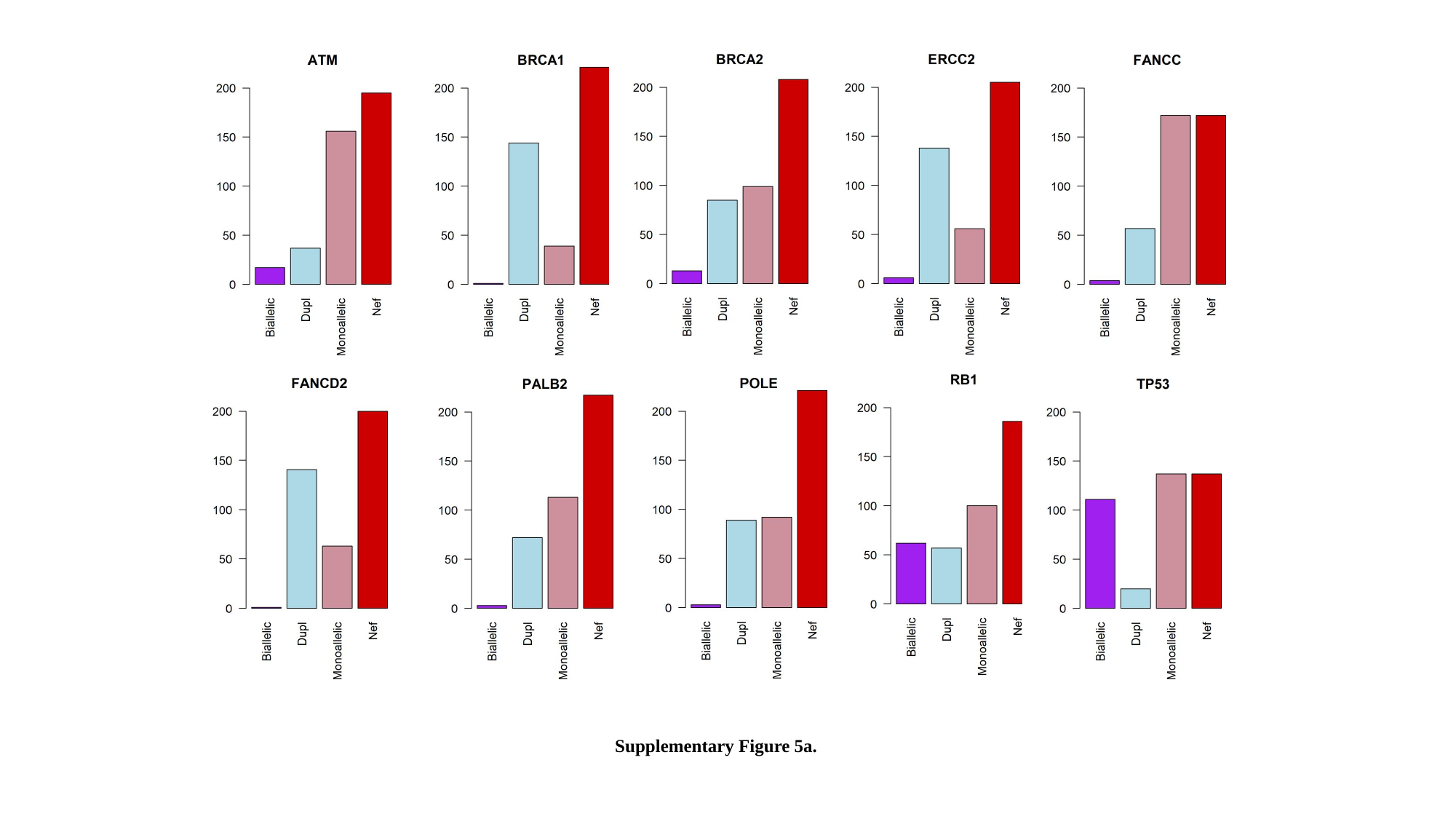

Supplementary Figure 5a.

## Slide 7
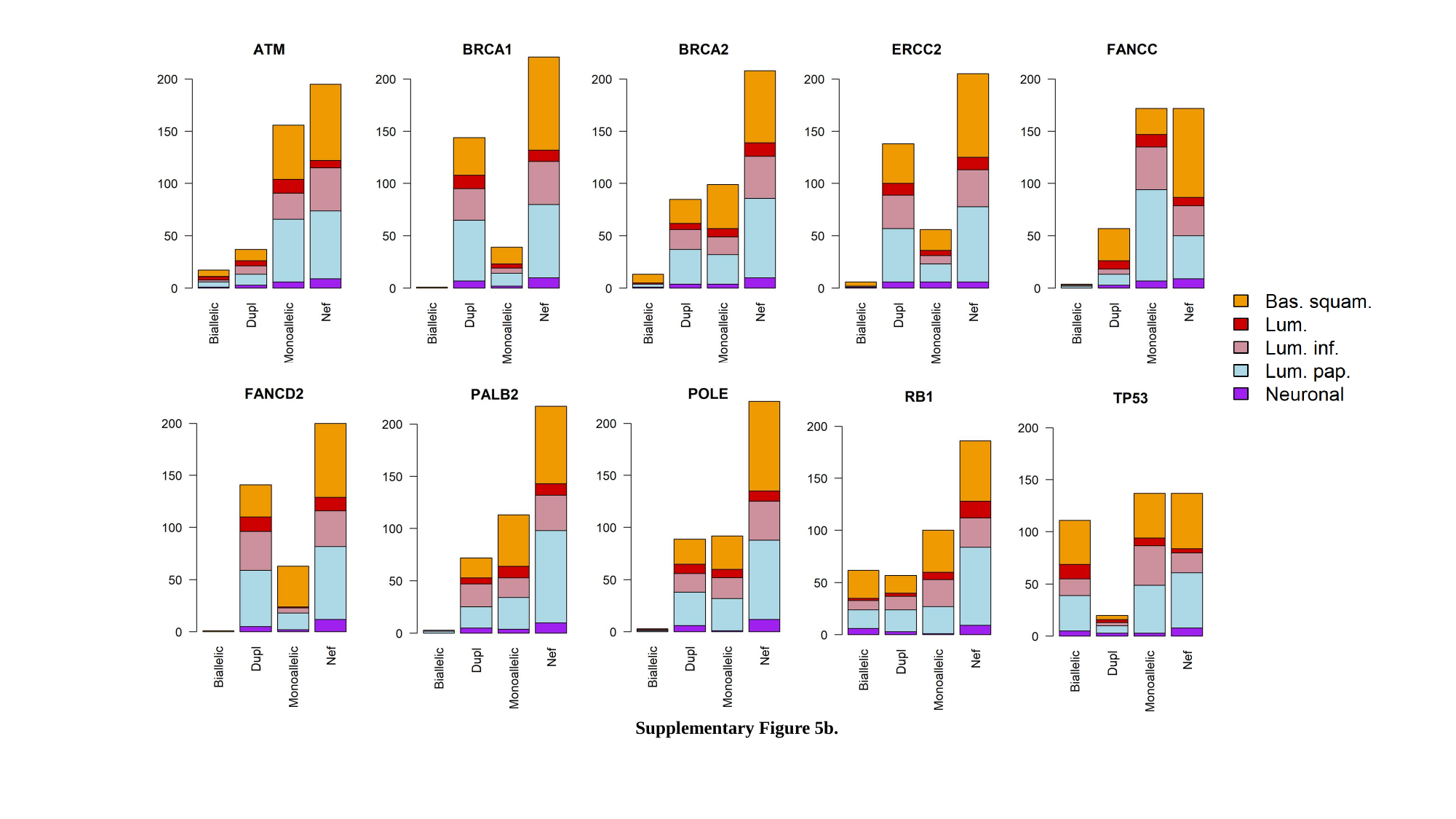

Supplementary Figure 5b.

## Slide 8
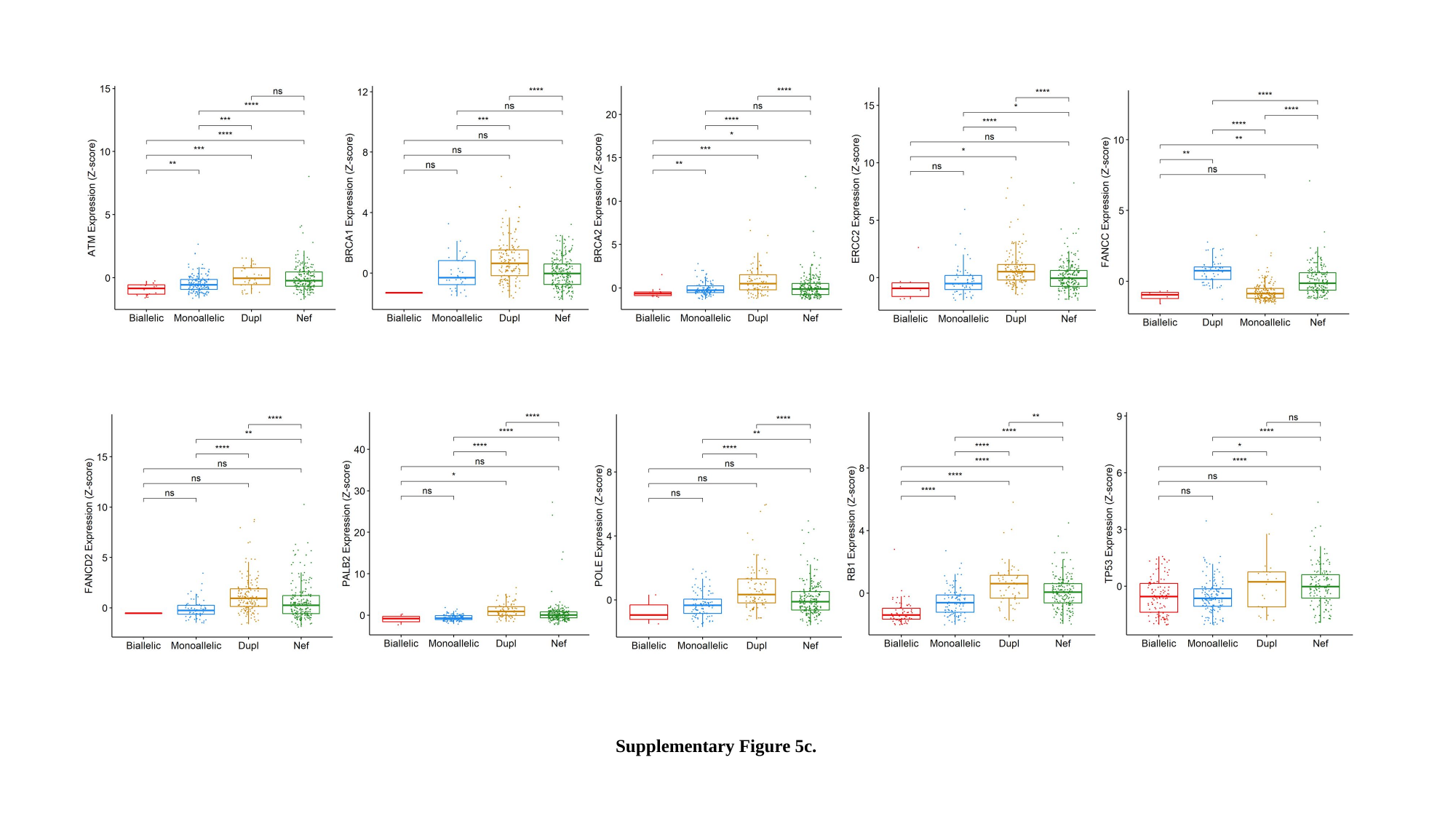

Supplementary Figure 5c.

## Slide 9
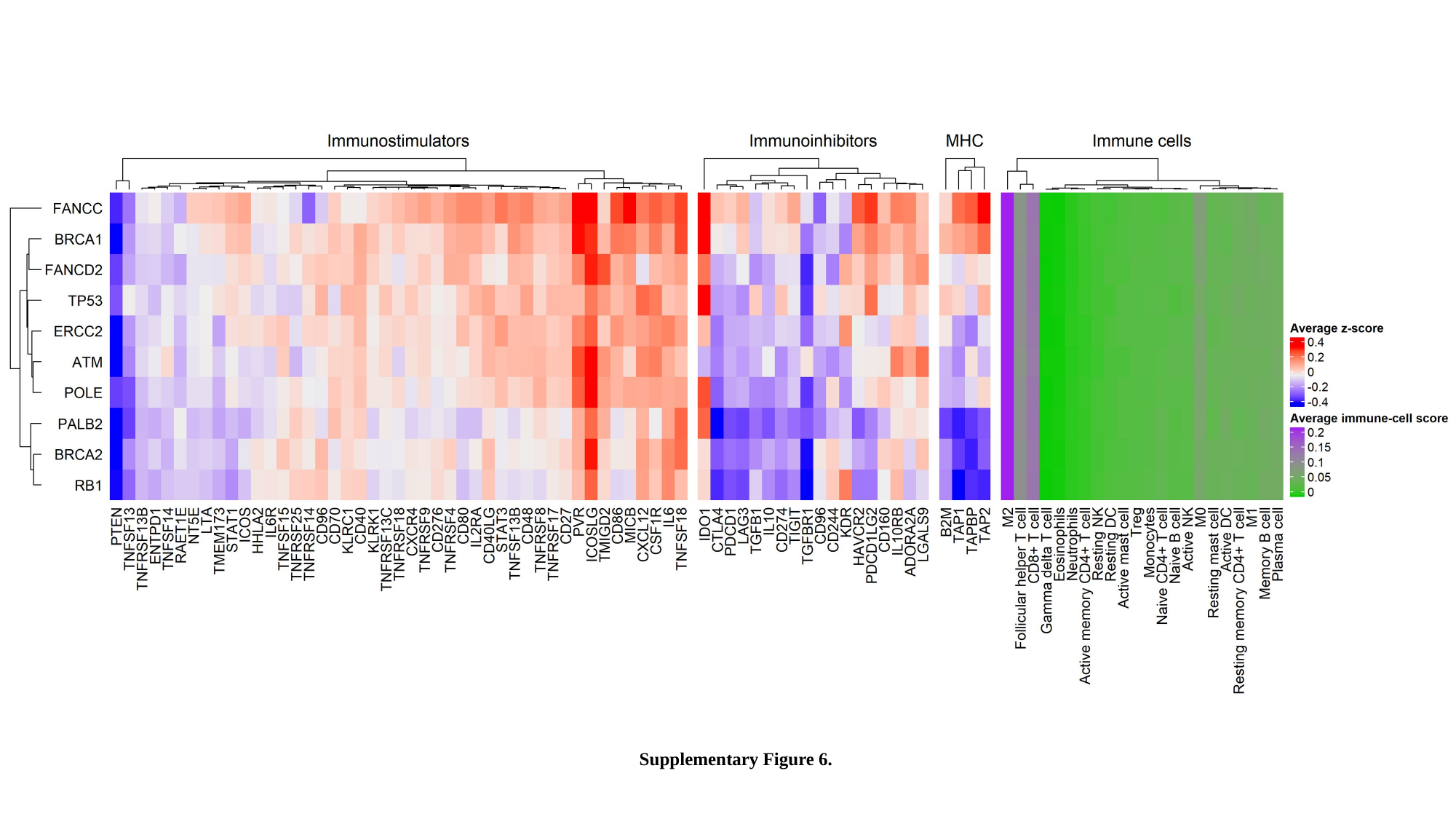

Supplementary Figure 6.

## Slide 10
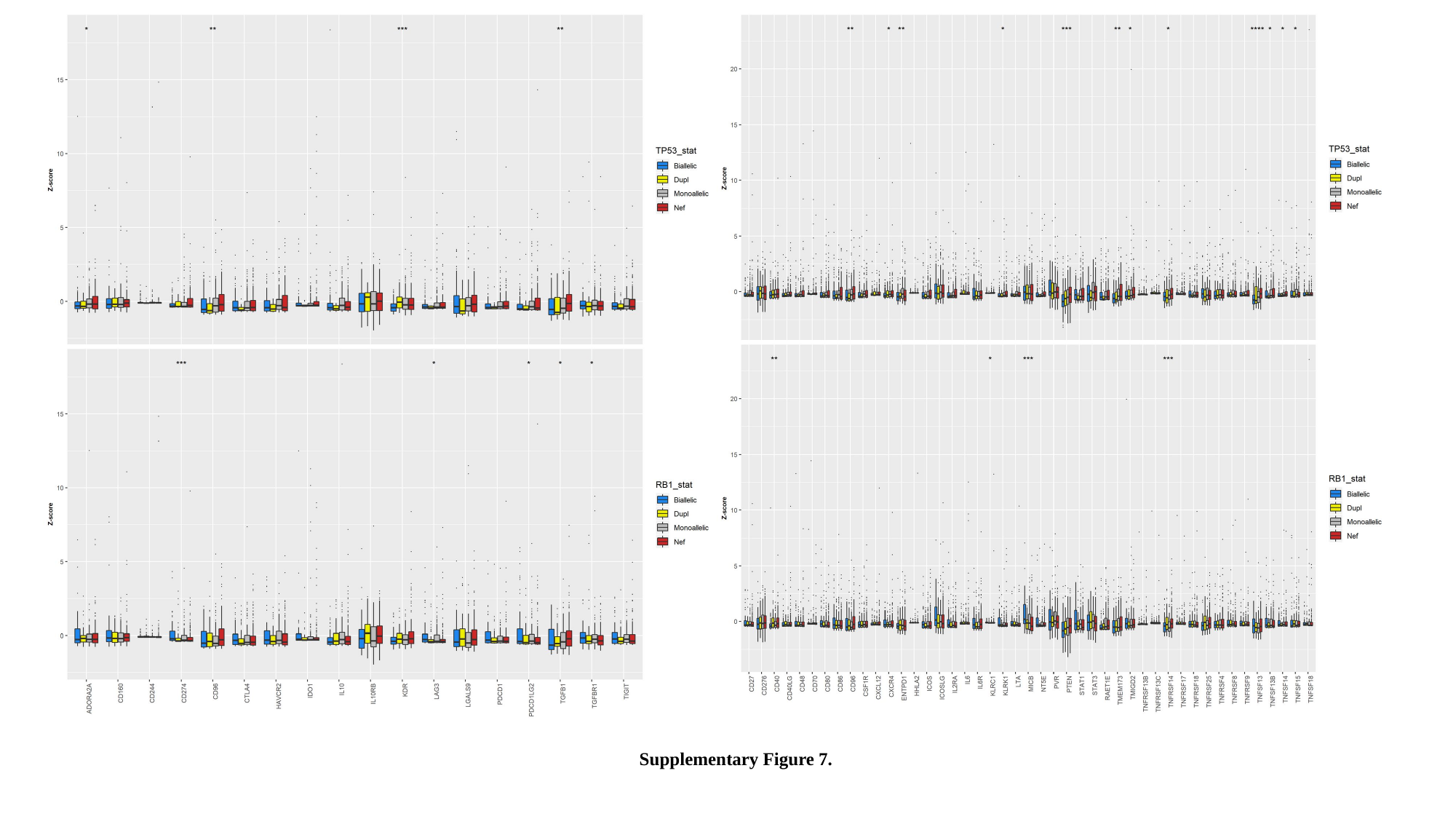

Supplementary Figure 7.

## Slide 11
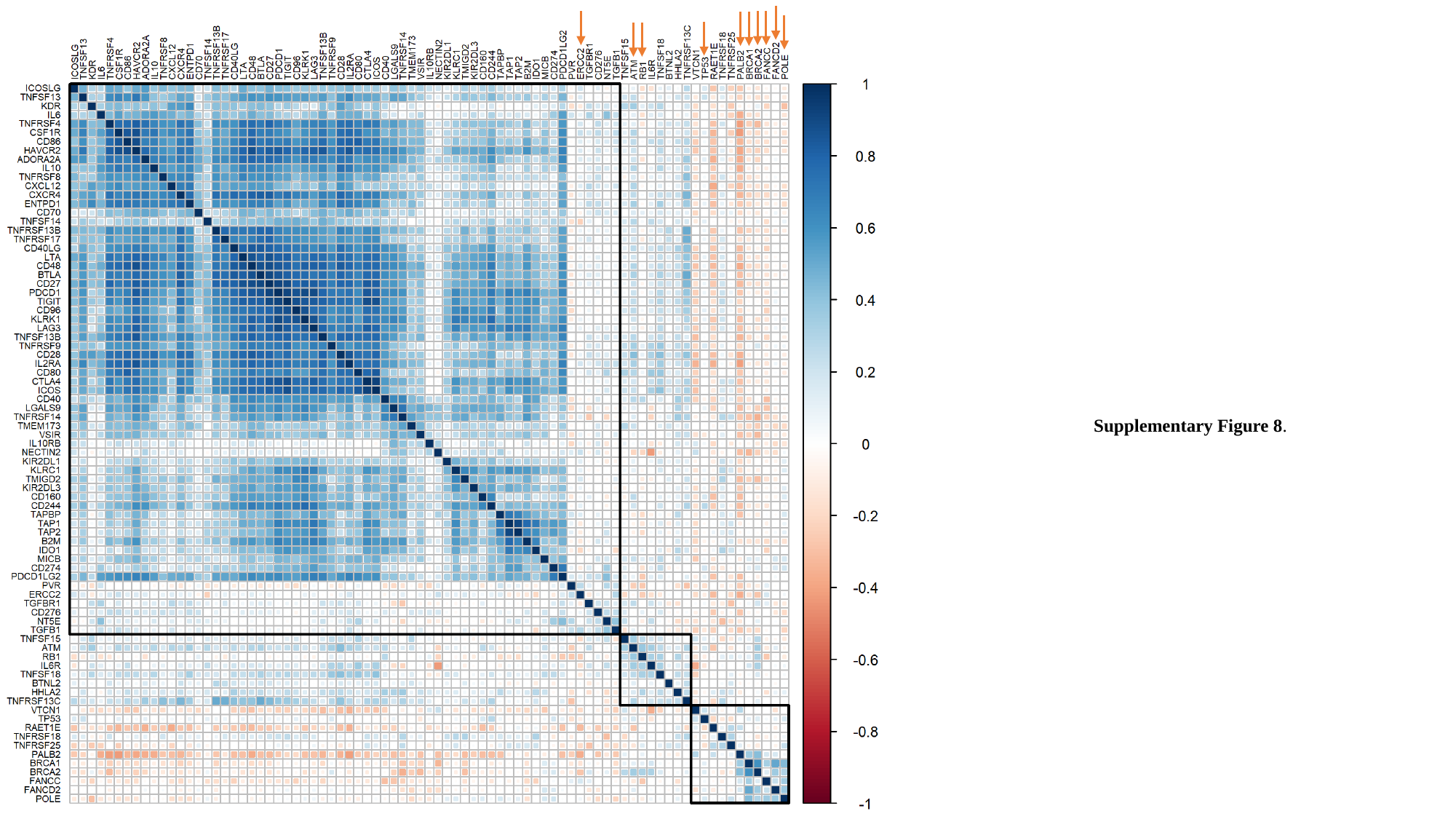

Supplementary Figure 8.

## Slide 12
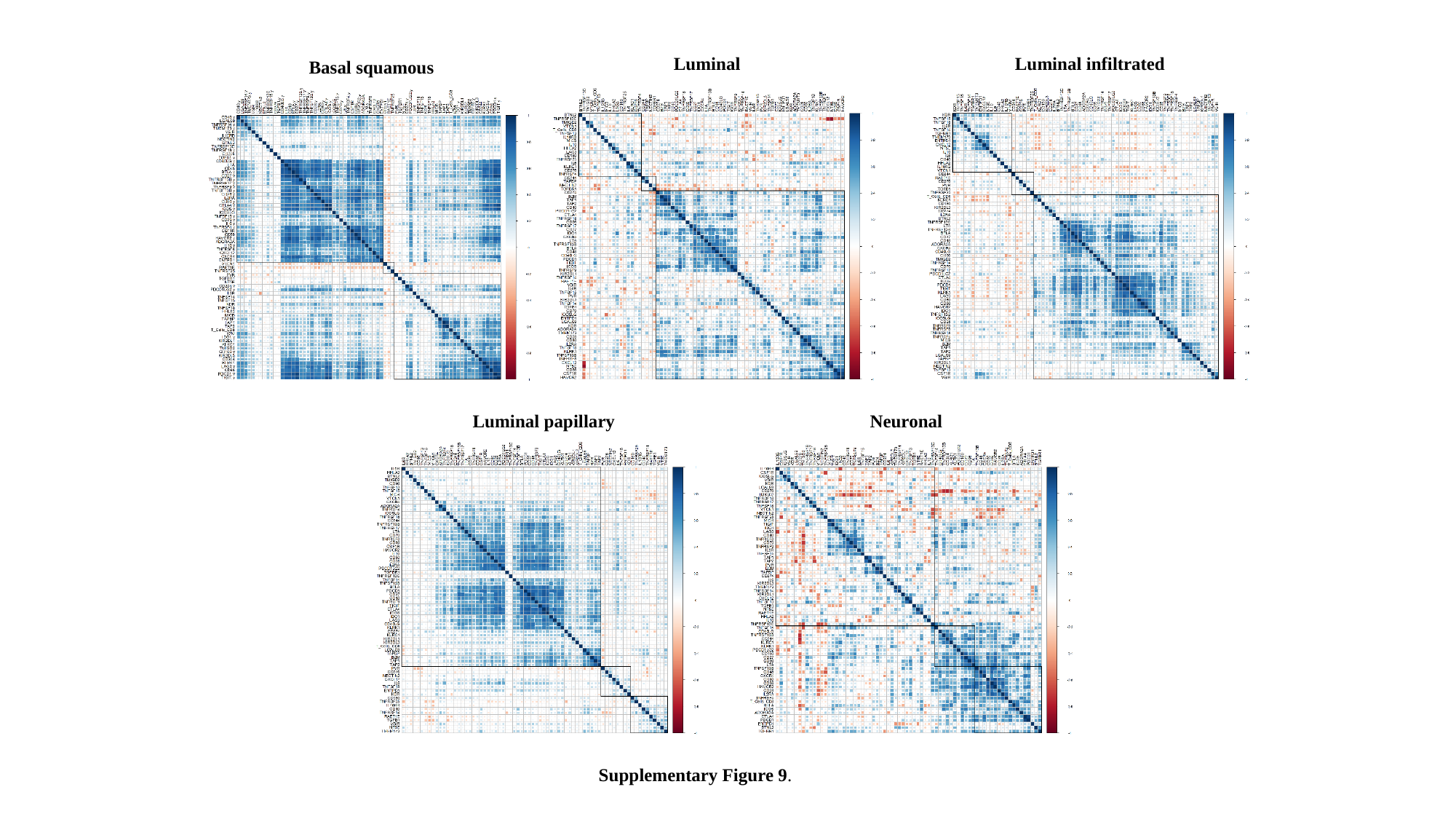

Luminal
Luminal infiltrated
Basal squamous
Luminal papillary
Neuronal
Supplementary Figure 9.

## Slide 13
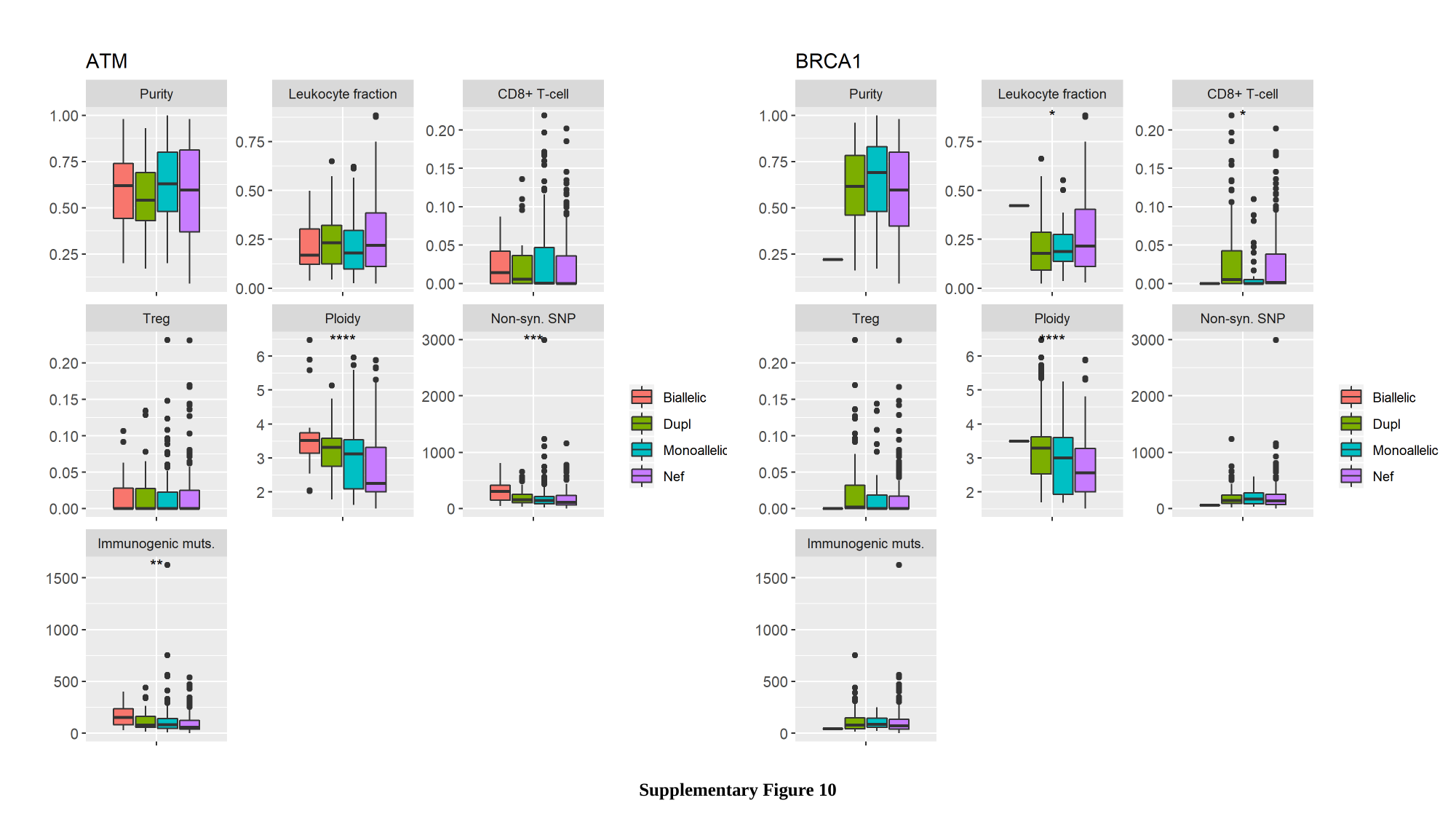

Supplementary Figure 10

## Slide 14
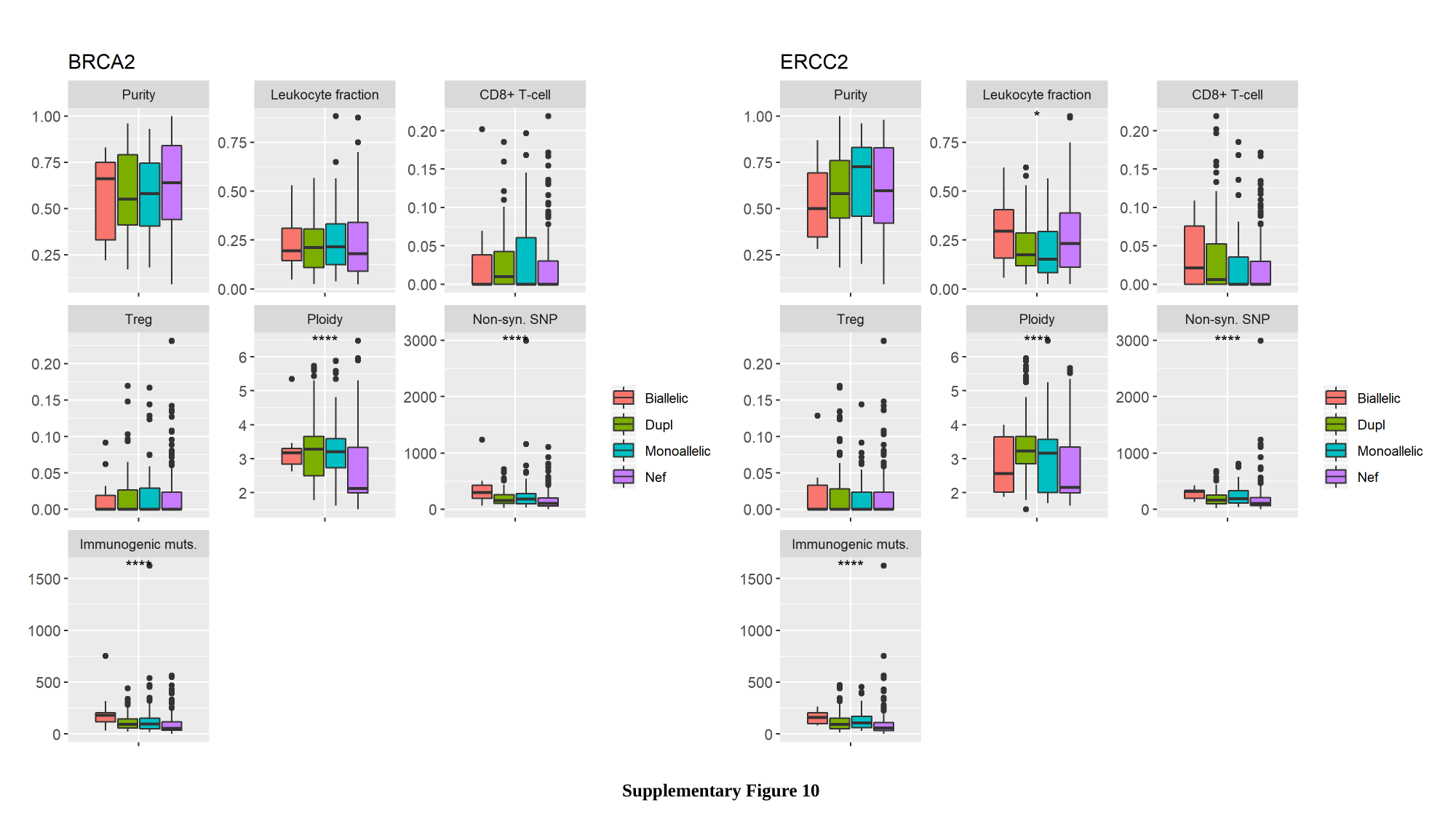

Supplementary Figure 10

## Slide 15
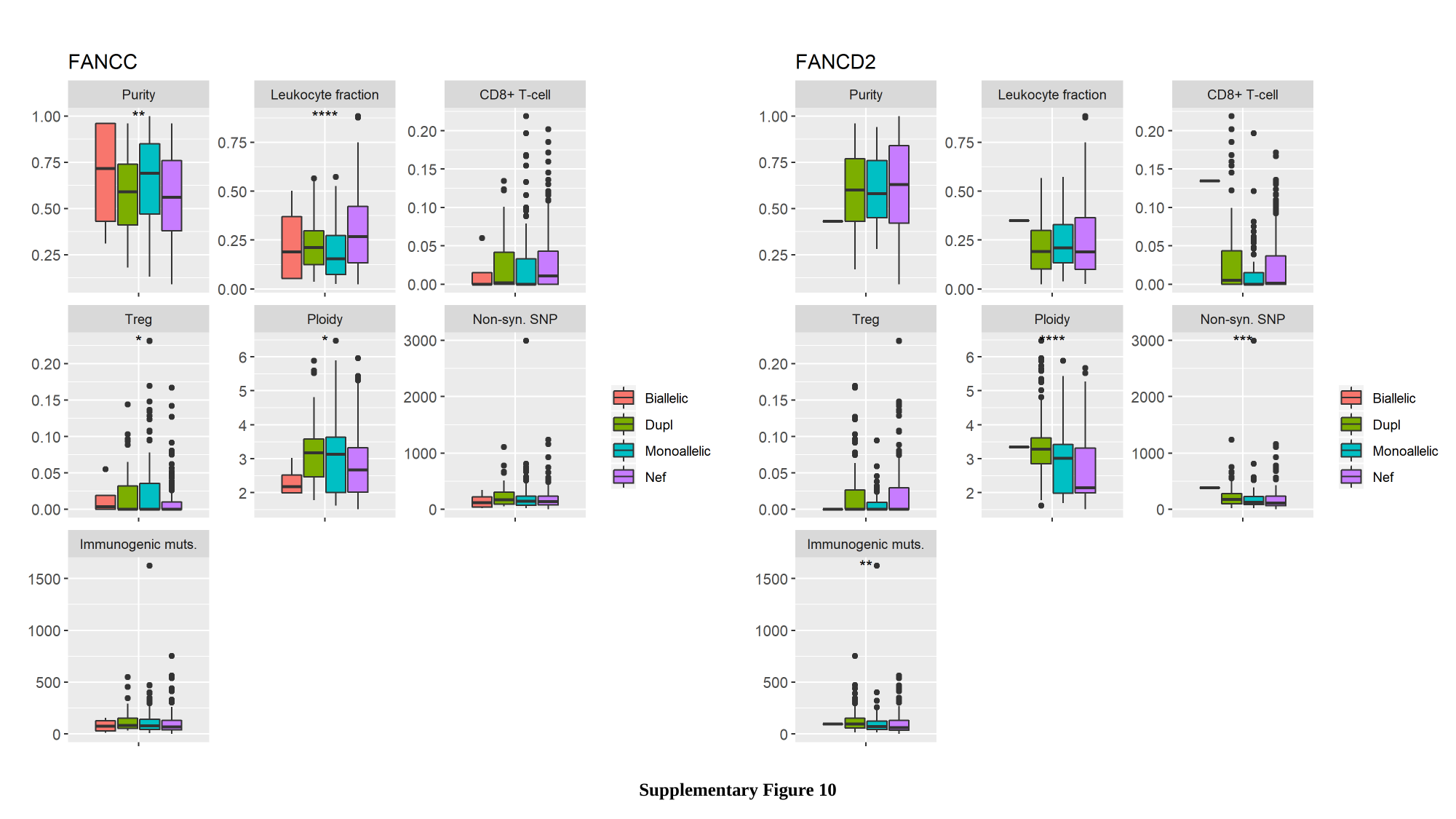

Supplementary Figure 10

## Slide 16
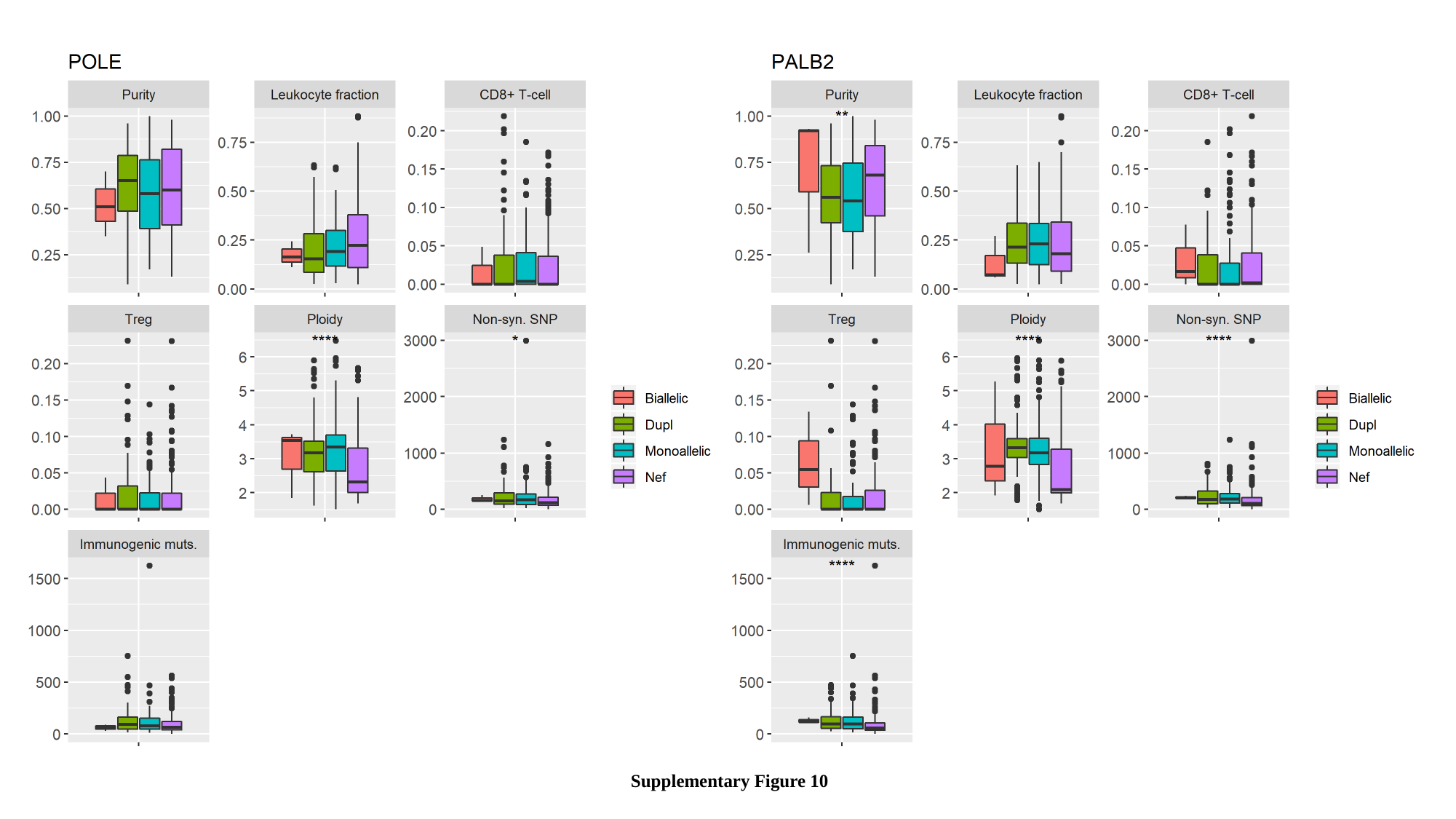

Supplementary Figure 10

## Slide 17
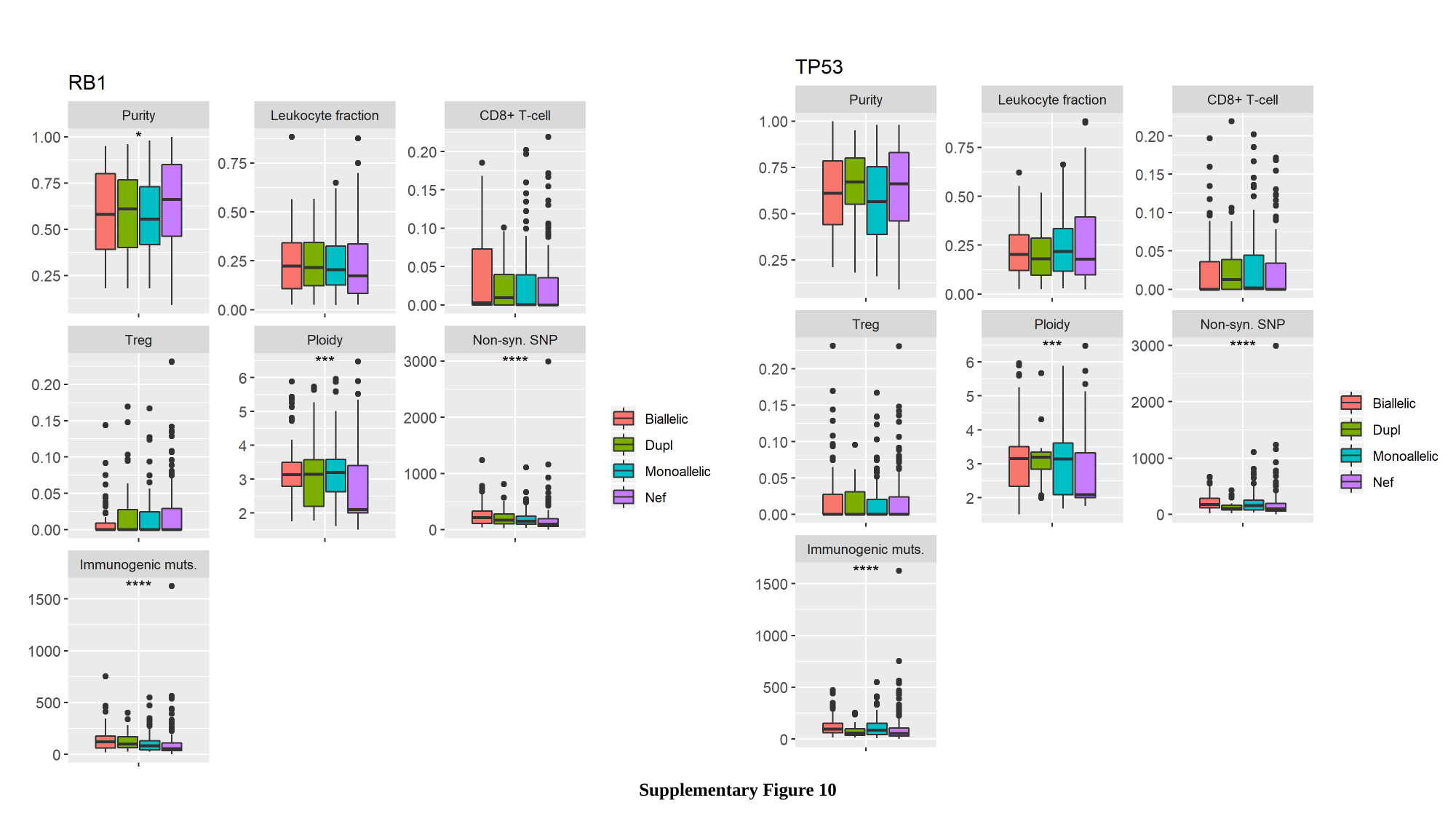

Supplementary Figure 10
